# Quinoline-Based Organic Frameworks with Divergent Photocatalytic Properties for Hydrogen Production and Furfuryl Alcohol Oxidation

**DOI:** 10.1021/acsaenm.5c00044

**Published:** 2025-03-28

**Authors:** Miguel Sánchez-Fuente, Emmanuel N. Musa, Ankit K. Yadav, Kyle T. Smith, Christopher N. Young, Alicia Moya, Xavier Solans-Monfort, Kyriakos C. Stylianou, Rubén Mas-Ballesté

**Affiliations:** ± Department of Inorganic Chemistry (Module 7), Facultad de Ciencias, 16722 Universidad Autónoma de Madrid, Madrid 28049, Spain; § Department of Chemistry (Module 7), Facultat de Ciencies, 16719 Universitat Autònoma de Barcelona, Bellaterra 08191, Spain; √ Materials Discovery Lab (MaD Lab), Department of Chemistry, 2694 Oregon State University, Corvallis, Oregon 97331, United States; † Analytical and Development Laboratories, HP Inc., 1000 NE Circle Blvd., Corvallis, Oregon 97330, United States; ¥ Institute for Advanced Research in Chemical Sciences (IAdChem), 16722 Universidad Autónoma de Madrid, Madrid 28049, Spain

**Keywords:** photocatalysis, organic materials, conjugated microporous polymers, hydrogen evolution reaction, furfuryl alcohol oxidation

## Abstract

The Sonogashira coupling reaction between 1,3,5-triethynylbenzene and two quinoline-based building units—6-iodo-2-(4-iodophenyl)-4-phenylquinoline or 6-bromo-2-(5-bromopyridin-2-yl)-4-phenylquinoline—resulted in the synthesis of conjugated materials designated as quinoline-based organic frameworks (QOFs), specifically **QOF1** and **QOF1-N**. These materials exhibited comparable structural and photophysical properties and demonstrated their utility in photocatalysis, including hydrogen evolution reactions (HER) and the selective oxidation of furfuryl alcohol to 5-hydroxy-2­(5*H*)-furanone. Interestingly, **QOF1-N** outperformed **QOF1** in the photocatalytic oxidation of furfuryl alcohol via singlet oxygen (^1^O_2_), whereas **QOF1** exhibited superior efficiency in HER. The divergent photocatalytic activity of these frameworks was attributed to the differences in their electronic structures, which were analyzed using a combination of experimental techniques and theoretical modeling. These findings highlight the potential of tailored QOFs for diverse photocatalytic applications, emphasizing the role of structural and electronic tuning in enhancing catalytic activity.

## Graphical abstract

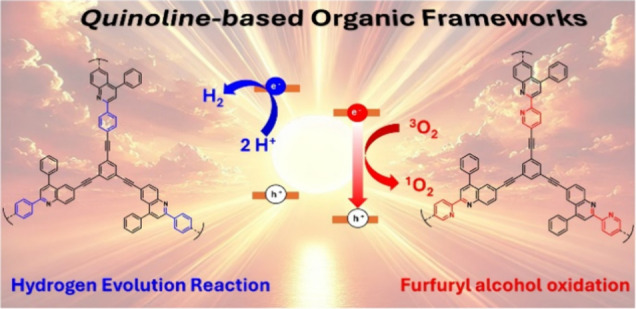

## Introduction

The transition to a greener, renewable production model has emerged as a significant challenge for science over the past few decades. Addressing the heavy reliance on petroleum-based fuels and identifying sustainable sources for industrial chemical precursors demands innovative approaches with minimal or, ideally, zero carbon footprints. Hydrogen, as a clean energy carrier, stands out as a promising alternative to conventional energy sources. However, its widespread adoption is hindered by the unfavorable energy balance associated with large-scale industrial production.^1^ Simultaneously, for synthesizing small organic precursors such as C4, C5, and C6 hydrocarbons—including compounds like furanone^2^—the focus has shifted toward developing sustainable pathways for their production. Biomass valorization offers a viable solution, presenting an alternative route to extracting them from nonrenewable sources. These dual challenges highlight the critical need for transformative strategies in energy and chemical synthesis to drive the transition toward a more sustainable future.

Photocatalysis has emerged as a promising solution for addressing energy and chemical synthesis challenges.^3–9^ Among the leading candidates for heterogeneous photocatalysts are reticular organic materials such as Covalent Organic Frameworks^10–12^ (COFs) and their related amorphous materials, including Covalent Triazine Frameworks (CTFs)^13,14^ or Conjugated Microporous Polymers (CMPs).^15,16^ These materials stand out due to their structural versatility, allowing the incorporation of diverse functional building blocks. These frameworks form predictable structures and topologies by linking predetermined organic building blocks, enabling precise modulation of their photophysical properties and fine-tuning of their photocatalytic activity. CMPs, in particular, exhibit extended π-conjugation within amorphous yet layered structures, providing a high degree of electron mobility. This unique combination of features makes CMPs attractive candidates for photocatalytic applications. Moreover, their photocatalytic activity can be strategically tailored by adjusting the monomer composition, polymerization degree, and reaction conditions.^15,17,18^

Photocatalytic processes typically proceed through one of two primary mechanisms: energy transfer or electron transfer,^19^ each dictating distinct reaction pathways. Regardless of the mechanism, the process begins with the excitation of the photocatalyst. In the electron transfer mechanism, the excited photocatalyst acts as a strong oxidizing or reducing agent, driving redox reactions with organic substrates.^20^ This interaction leads to the generation of radical intermediates, which subsequently transform into the final products.^21^ Conversely, the energy transfer mechanism involves directly transferring absorbed energy from the photocatalyst to the reactants, activating them, and initiating the desired chemical transformation.^22^ Despite the importance of these mechanisms, the structural features that govern the choice of one pathway over the other remain an unresolved question. This critical aspect of photocatalysis has received limited attention in the literature, leaving significant gaps in understanding their properties. For instance, recent studies suggest that certain structural features, such as imine linkages commonly found in COFs, can act as barriers to electronic conjugation within organic materials.^23^ Such disruptions in conjugation may play a pivotal role in influencing the preferred photocatalytic mechanism, yet the broader implications of this phenomenon are still underexplored. Also, the structure and morphology of imine-based COFs play a key role in determining their photocatalytic behavior in light-driven oxidation reactions.^24^ In addition, even minor modifications to the building blocks used in their synthesis—such as the presence of a sulfur atom that differentiates triphenylamine from phenyl phenothiazine fragments—can significantly impact the photocatalytic efficiency of the resulting porous organic materials.^41^ Understanding the influence of these structural and compositional factors is essential for advancing the design and synthesis of efficient organic heterogeneous photocatalysts. Such insights will not only accelerate the development of sustainable hydrogen production technologies but also enable the precise synthesis of industrially relevant chemicals from renewable biomass feedstocks, contributing to a greener chemical industry.

To gain insights into the design principles governing the photocatalytic pathways of CMPs, in this work, we report the synthesis of two novel organic materials incorporating distinct quinoline-based photoactive moieties: 2,4-diphenyl quinoline or 4-phenyl-2-(pyridine-2-yl)­quinoline. These moieties are linked through alkyne (C (sp)—C (sp)) bonds via Sonogashira coupling (Scheme 1), forming Quinoline-based Organic Frameworks (QOFs), designated as **QOF1** and **QOF1-N**. Recent studies have demonstrated that integrating quinoline moieties into covalent frameworks enhances photocatalytic activity.^25,26^ Building on this foundation, we investigate how a simple substitution of a phenyl group with pyridine in the QOF materials impacts photocatalytic activity across two environmentally significant reactions: hydrogen evolution reaction (HER) and furfuryl alcohol oxidation. HER typically involves electron transfer to water protons facilitated by a metallic cocatalyst, such as platinum (Pt).^27–29^ In contrast, furfuryl alcohol oxidation, a valuable transformation for producing biomass-derived chemical precursors, relies on oxygen activation—commonly mediated through energy transfer.^30,31^ Through a comparative study of **QOF1** and **QOF1-N** in these electron- and energy-transfer-driven photocatalytic processes, we elucidate the effects of subtle differences in their electronic structures. Our findings reveal how these structural variations influence photocatalytic behavior, offering critical insights into designing organic materials with tailored functionalities.

**Scheme 1:**
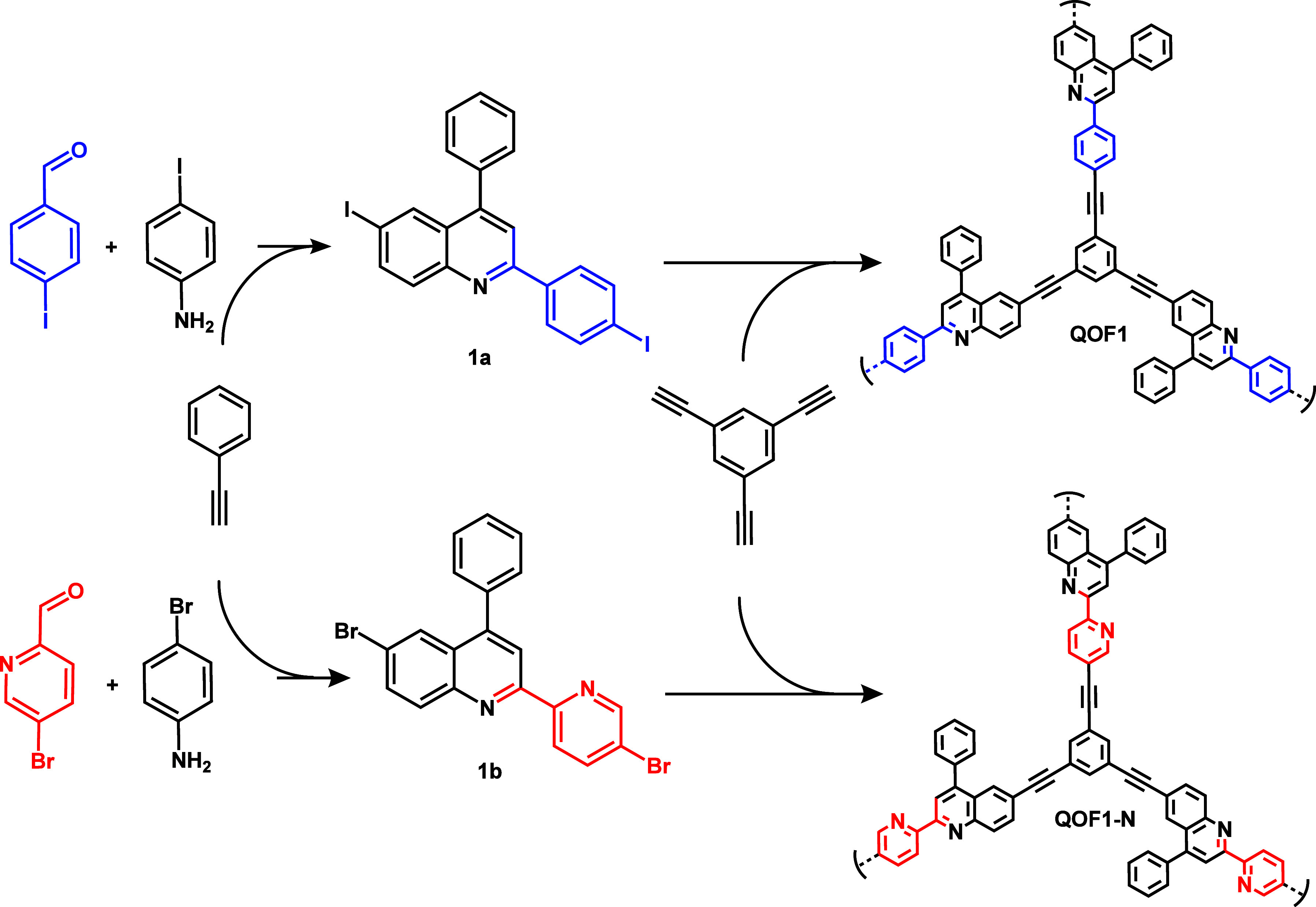
Synthetic Approach for **QOF1** and **QOF1-N** Materials

## Results and Discussion

### Synthesis and Characterization of Materials

The quinoline-based molecular precursors for each material were synthesized using Povarov′s reaction, involving the condensation of 4-halide-substituted benzaldehydes, anilines, and phenylacetylene. For the first precursor, 6-iodo-2-(4-iodophenyl)-4-phenylquinoline (**1a**), 4-iodobenzaldehyde, 4-iodoaniline, and phenylacetylene were reacted in the presence of scandium triflate (Sc­(OTf)_3_) as catalyst in toluene (Scheme S1). Similarly, 6-bromo-2-(5-bromopyridin-2-yl)-4-phenylquinoline (**1b**) was synthesized using 5-bromopinacolaldehyde, 4-bromoaniline and 4-phenylacetylene under comparable conditions (Scheme S2). The selection of iodo- or bromo-derivatives was guided by the availability of commercial precursors. The reaction methodology was adapted from previously reported protocols in the literature,^32^ ensuring reproducibility. The resulting products were characterized by ^1^H NMR, ^13^C NMR, and FT-IR spectroscopies (Figures S1, S2 and S4). The spectral data was consistent with those of structurally related systems reported in the literature,^32^ confirming the successful synthesis of the quinoline-based precursors (detailed characterization data available in Supporting Information and Figure 1).

**Figure 1:**
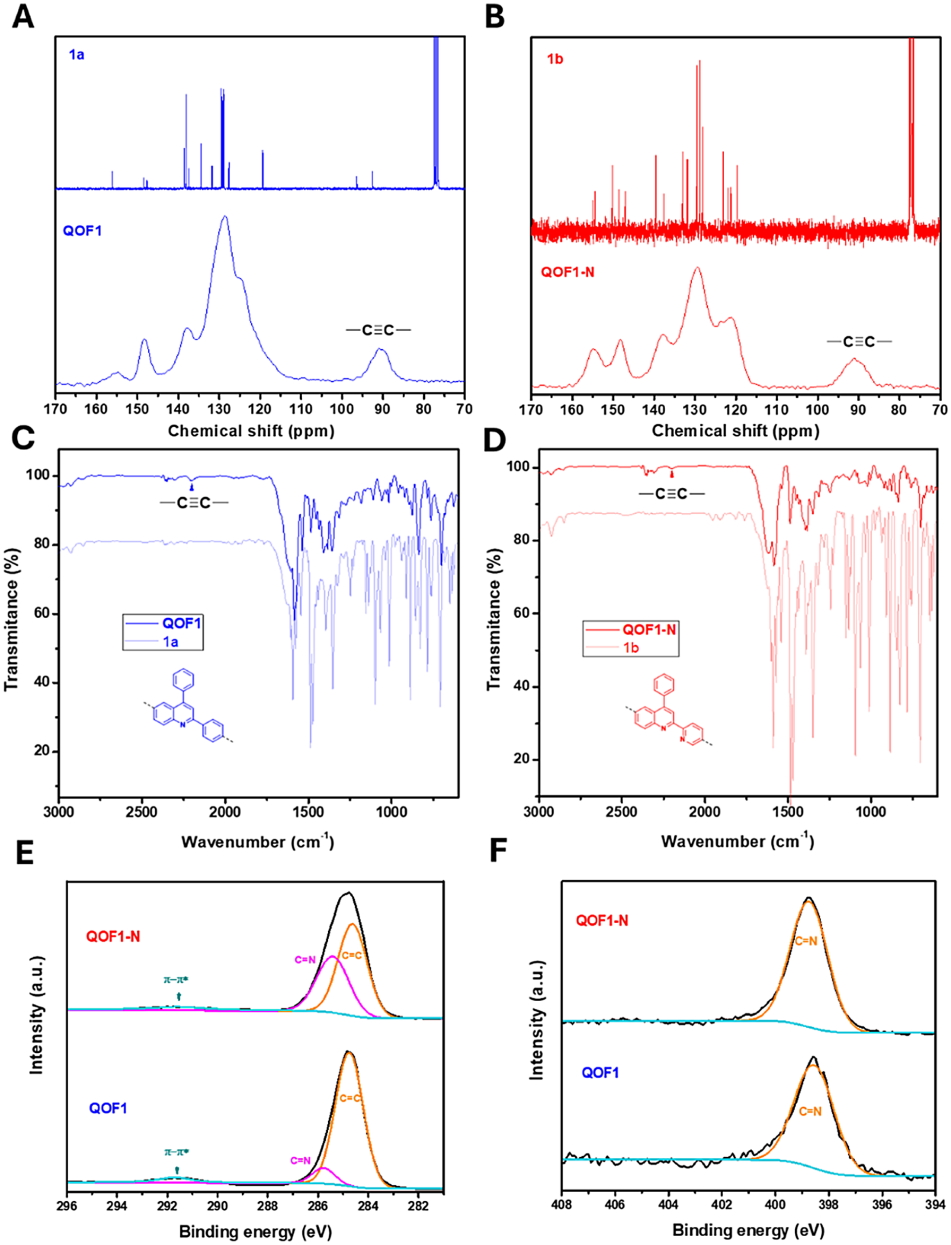
Solid State-CP/MAS ^13^CNMR of A) **QOF1**(blue) and B) **QOF1-N** (red) stacked with their respective quinoline building units **1a** and **1b** ^13^C NMR in solution. FTIR Spectra in KBr of C) **QOF1** (blue) and D) **QOF1-N** (red) overlapped with their respective quinoline building units **1a** and **1b**. E) XPS C 1s region and F) XPS N 1s region of **QOF1** and **QOF1-N** spectra with corresponding fitted peaks.

The materials, **QOF1** and **QOF1-N**, were synthesized by coupling each dihalogenated quinoline monomer with 1,3,5-triethynylbenzene using a Sonogashira-type coupling reaction (Scheme S3 and Table S1). This well-established methodology has been widely employed for the synthesis of diverse CMPs.^33^ The resulting materials were isolated as fine yellow powders, exhibiting complete insolubility in all common solvents, indicative of their robust cross-linked structures (Scheme 1). Comprehensive characterization of **QOF1** and **QOF1-N** is detailed in the subsequent paragraphs.

Initial insights into the chemical identity of the materials were obtained through solid-state ^13^C NMR spectroscopy using cross-polarization combined with magic angle spinning (CP/MAS ^13^C NMR). Peaks at 155 and 148 ppm were attributed to the conjugated —C = N— quaternary carbons present in both frameworks. Notably, in **QOF1-N**, the peak at 155 ppm was more intense, reflecting the additional —C = N— group introduced by the pyridine substituent (see Figure 1B). The alkyne linkers’ C­(sp) quaternary carbons were observed around 91 ppm in both materials, confirming the proposed alkyne-linked structures (Figure 1A and 1B). Comparison with the ^13^C NMR spectra of the molecular quinoline precursors in solution revealed similar patterns for the quinoline moieties and (Figure S3B). It is important to note that the ^13^C NMR spectrum of the di-iodide precursor **1a** contains two signals at 96 and 92 ppm corresponding to the carbon atoms bonded to iodide, which could be confused with the C­(sp) signal observed in the solid-state spectrum of **QOF1**. The FTIR spectra of both materials also provided valuable structural information, showing characteristic peaks at 2202 cm^–1^ corresponding to the alkyne linkers, albeit with low intensity (Figure S3A). Most of the characteristic signals from their respective quinoline precursors were retained in both materials. For **QOF1**, bands at 1622, 1588, 1570, 1540, and 1485 cm^–1^, derived from quinoline **1a**, were present in the material’s structure. Similarly, for **QOF1-N**, characteristic bands at 1620, 1589, 1570, 1540, and 1489 cm^–1^ were observed, matching those from the quinoline **1b** spectrum (Figure 1C and 1D). In addition, the aromatic C = C vibration at 1468 and 1469 cm^–1^ in **1a** and **1b**, respectively, shift to higher wavenumbers upon conversion of C­(sp^2^)—X (X = I for 1a and Br for 1b) to C­(sp^2^)—C­(sp) in the final materials.

X-ray photoelectron spectroscopy (XPS) analysis of the **QOF1** and **QOF1-N** materials provided valuable insights into their chemical composition and electronic structure (Figure S5). The C 1s spectra of **QOF1-N** exhibited a slightly broader signal compared to **QOF1**, indicating subtle differences in their electronic environments. A detailed spectral fit revealed three main contributions in the C 1s region for both materials (Figure 1E). The two dominant carbon signals, corresponding to C = C and C = N bonds, were observed at 284.8 and 286.5 eV for **QOF1** and at 284.8 and 286.2 eV for **QOF1-N**, respectively. The slight changes in the C = N signal in **QOF1** and **QOF1-N** are attributed to the additional nitrogen atoms present not only in the quinoline structure but also in the pyridine moiety. Furthermore, both materials exhibited π-π* stacking interactions in the 290–293 eV range, further confirming the laminar, conjugated structure of these materials. The N 1s spectra of both materials displayed a single peak at 398.7 eV, corresponding to the sp^2^ nitrogen atoms in the quinoline and bipyridine fragments. This binding energy, consistent with literature values,^26^ reflects the indistinguishable chemical environments of the nitrogen atoms in both structural motifs (Figure 1F).

The photophysical properties of **QOF1** and **QOF1-N** were compared through UV–visible absorption and emission spectra. Both materials exhibited strong absorption in the range of 300–440 nm (see Figure 2A). Emission spectra showed that **QOF1** emitted at a slightly higher energy, with a maximum at 496 nm (λ_exc_ = 385 nm), whereas **QOF1-N** emitted at 504 nm (λ_exc_ = 385 nm) (see Figure 2B). Applying the Kubelka–Munk transformation to the absorption data, the direct band gaps for both materials were calculated to be 2.76 eV (see Figures S6 and S7 and Table S2). The valence band (VB) positions were determined from the XPS spectra in the region from 0 to 25 eV range relative to the Normal Hydrogen Electrode (NHE) (Figure 2C). The VB edge positions were found to be −5.62 eV for **QOF1** and −5.98 eV for **QOF1-N**, corresponding to 1.22 and 1.58 eV vs NHE, respectively. The conduction band (CB) positions were calculated by adding the bandgap energy to the VB positions, resulting in values of −2.86 eV for **QOF1** and −3.22 eV for **QOF1-N** (−1.54 and −1.18 eV vs. NHE). Notably, the CB position of **QOF1-N** was slightly downshifted by 0.36 eV relative to **QOF1** (Figure 2D).

**Figure 2:**
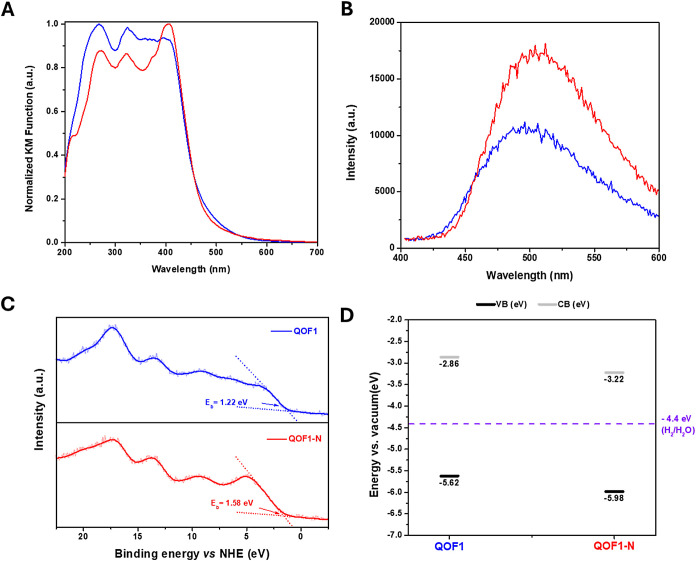
Photophysical characterization of **QOF1** (blue) and **QOF1-N** (red) materials. A) UV–vis Absorption spectra. B) Photoluminescence emission spectra (λ_exc_ = 385 nm). C) XPS spectrum onset of **QOF1** (blue) and **QOF1-N** (red) for their respective CB calculation. D) VB and CB diagram for **QOF1** and **QOF1-N**.

The thermal stability of both materials was evaluated using thermogravimetric analysis (TGA) under argon atmosphere. Both **QOF1** and **QOF1-N** exhibit good stability at low and moderate temperatures. A weight loss at around 80 °C, corresponding to solvent desorption, was 8% for **QOF1-N** and 5% for **QOF1**. **QOF1** maintained its structural integrity up to 465 °C, whereas **QOF1-N** remained stable up to 336 °C (see Figure S8). Scanning electron microscopy (SEM), analysis revealed that both **QOF-1** and **QOF1-N** experience the collapse of their laminar structures into hollow needle- and sphere-like morphologies, along with less defined morphologies (see Figures 3 and S9–S10). Neither material exhibited permanent porosity, as N_2_ and CO_2_ isotherms indicated, and no crystallinity was observed (Figure S11). As previously observed for CMPs,^34^ the lack of crystallinity and porosity is likely a result of highly irreversible covalent assembly, which prevents self-healing processes necessary for crystallization. Despite the absence of crystallinity and porosity, both materials exhibited photocatalytic activity.

**Figure 3:**
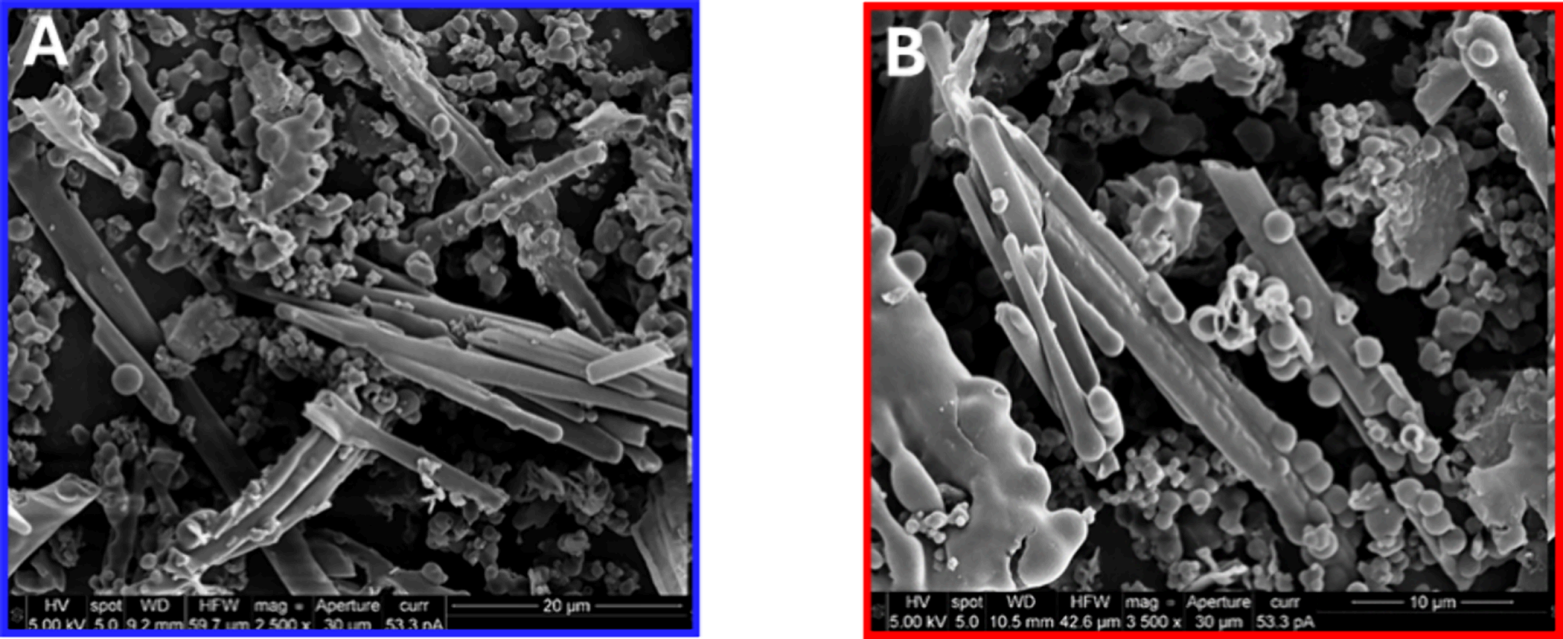
SEM images A) **QOF1** and B) **QOF1-N**.

### Catalytic Study

To evaluate the catalytic efficiency of **QOF1** and **QOF1-N** in photocatalytic electron transfer reactions, we tested both materials as photocatalysts for HER in the presence of triethylamine (TEA) as a sacrificial electron donor (see Figure 4). After optimizing the catalyst loading and reaction time for **QOF1** in the presence of Pt as cocatalyst (0.147 mg; 5.5 wt %) (see Figure S12), we fixed 2.5 mg of catalyst and 4 h of reaction time per catalytic experiment as standard reaction conditions. Without a cocatalyst, **QOF1** exhibited a modest HER rate of 195 μmol·h^–1^·g^–1^, while no detectable HER activity was observed for **QOF1-N**. However, introducing H_2_PtCl_6_ (for the generation of Pt nanoparticles) as a cocatalyst to facilitate electron transfer led to a significant increase in the HER rate of **QOF1**, reaching 761 μmol·h^–1^·g^–1^. In contrast, the HER activity of **QOF1-N** remained low even in the presence of Pt cocatalyst, only reaching 177 μmol·h^–1^·g^–1^. Despite the similarities in the photophysical properties of the two materials, their ability to catalyze photoinduced HER through electron transfer processes (see Scheme S4) differs significantly. Furthermore, the recyclability of **QOF1** was evaluated without adding more Pt cocatalyst, with each cycle revealing consistent HER rates over three cycles (Figure 4B). To further investigate the photocatalyst-cocatalyst system, we performed energy-dispersive X-ray spectroscopy (EDX) coupled with SEM on the **QOF1** material recovered postphotocatalysis. The EDX analysis confirmed the presence of Pt in the sample postphotocatalysis (Figure S13). This finding suggests a stable interaction between the cocatalyst and the photocatalyst, enabling its reuse across multiple cycles without reloading the cocatalyst. Additionally, SEM and ATR-FTIR analysis revealed that the morphology and chemical nature of the **QOF1** material were preserved (Figure S14 and S15). XPS analysis post-HER indicates that the photodeposition of Pt on **QOF1** remains primarily in its neutral form (Pt^0^), while **QOF1-N** exhibits a shift in the Pt 4f_7/2_ binding energy indicating the presence of both Pt^0^ and Pt^2+^.^35,36^ Additionally, while the N 1s spectra of **QOF1** remain consistent before and after the reaction (Figure S16), changes in the N 1s spectra of **QOF1-N** post-HER further suggests a different redox behavior of Pt/quinoline-pyridine moieties in **QOF1-N**.

**Figure 4:**
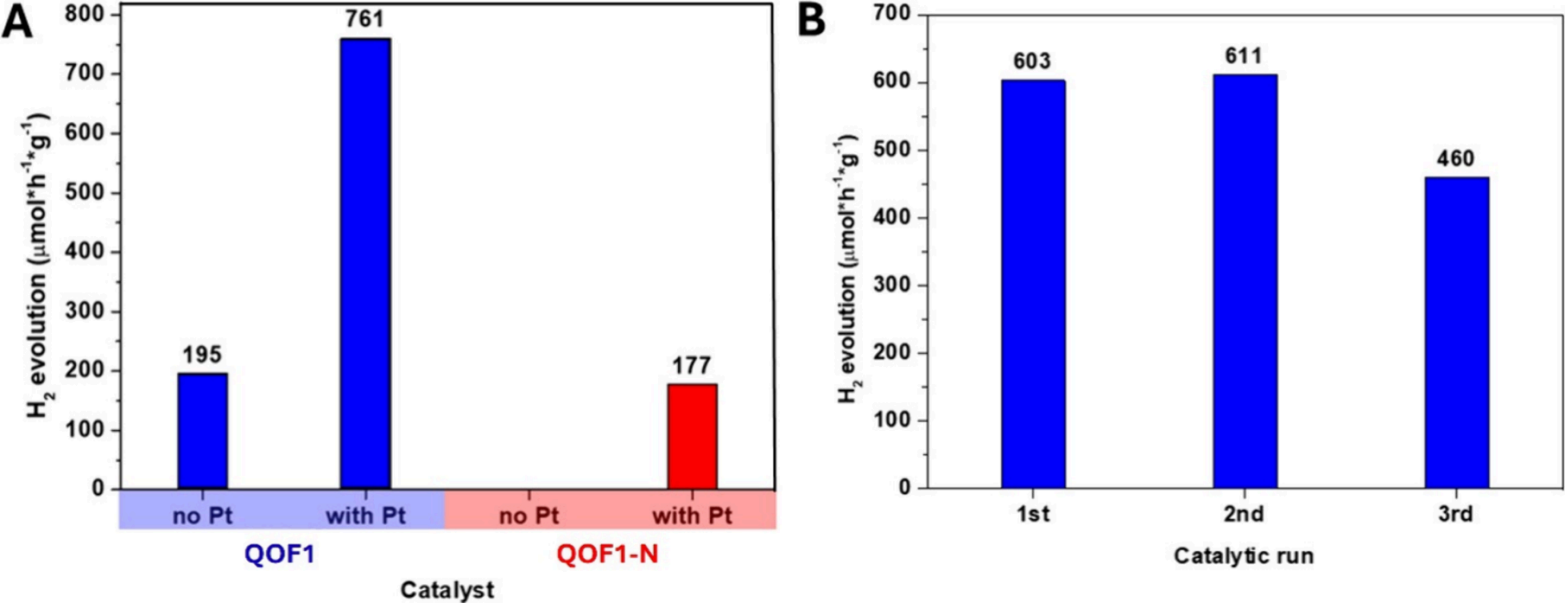
A) HER experiments for **QOF1** (blue) and **QOF1-N** (red) materials, using a 300 W Xe Lamp equipped with a > 420 nm cutoff filter over (4 h reaction time). B) Recyclability test of **QOF1** (2h per run).

We further explored the photocatalytic activity of these materials in the photooxidation of furfuryl alcohol (FA). Our results reveal that the catalysts are capable of oxidizing FA into 5-hydroxy-2­(5H)-furanone (5H5F) (Figure 5). This 4C lactone product is of practical significance, as it serves as a key precursor for various compounds of interest in organic synthesis, including maleic acid, 1,4-butanediol, or γ-butyrolactone.^2^ Additionally, it is a valuable intermediate for synthesizing chiral active compounds like (+)-Aflatoxin^37^ or (−)-Kainic acid.^38^ It has been reported that the oxidation of furfuryl alcohol proceeds via the formation of singlet oxygen (^1^O_2_) species through an energy transfer mechanism.^31^ However, this type of oxidation is often nonselective. It is commonly associated with furfuraldehyde and furanoic acid formation,^30^ but less frequently reported for 5H5F, whose synthesis offers more challenges.^5^ The difficulty in obtaining the 5H5F product from the direct oxidation of FA is due to the wide variety of photooxygenation byproducts derived from the photooxidation of furans and the required deformylation step.^31^ Interestingly, when a solution of FA in acetonitrile (MeCN) was photocatalytically oxidized using both **QOF1** and **QOF1-N**, the primary product formed was 5H5F, identified by ^1^H NMR (see Figures S18–S21) with no trace of furfuraldehyde as a side product. After optimizing the reaction time for **QOF1 and QOF1-N**, we fixed the standard reaction conditions at 30 min per catalytic experiment (see Figure S17). Notably, **QOF1-N** exhibited a significantly higher catalytic efficiency, achieving 84% conversion of FA into 5H5F within 30 min, compared to only 28% for **QOF1** under the same conditions (Scheme 2). Control experiments for FA oxidation in the absence of a catalytic material were carried out, with no observable conversion.

**Scheme 2:**
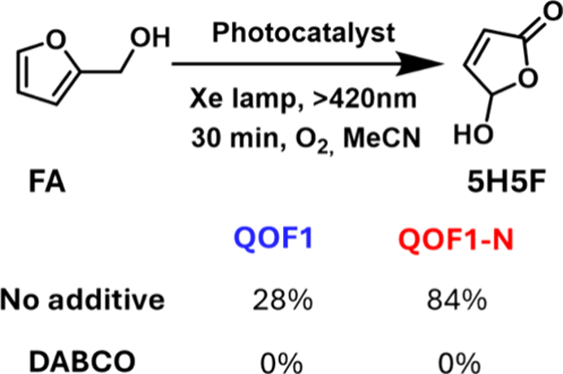
Photooxidation Experiments of Furfuryl Alcohol with **QOF1** and **QOF1-N** in the Presence and Absence of DABCO^a^

a The percentage values are the conversions measured by ^1^H-NMR peak integration from the crudes.

**Figure 5:**
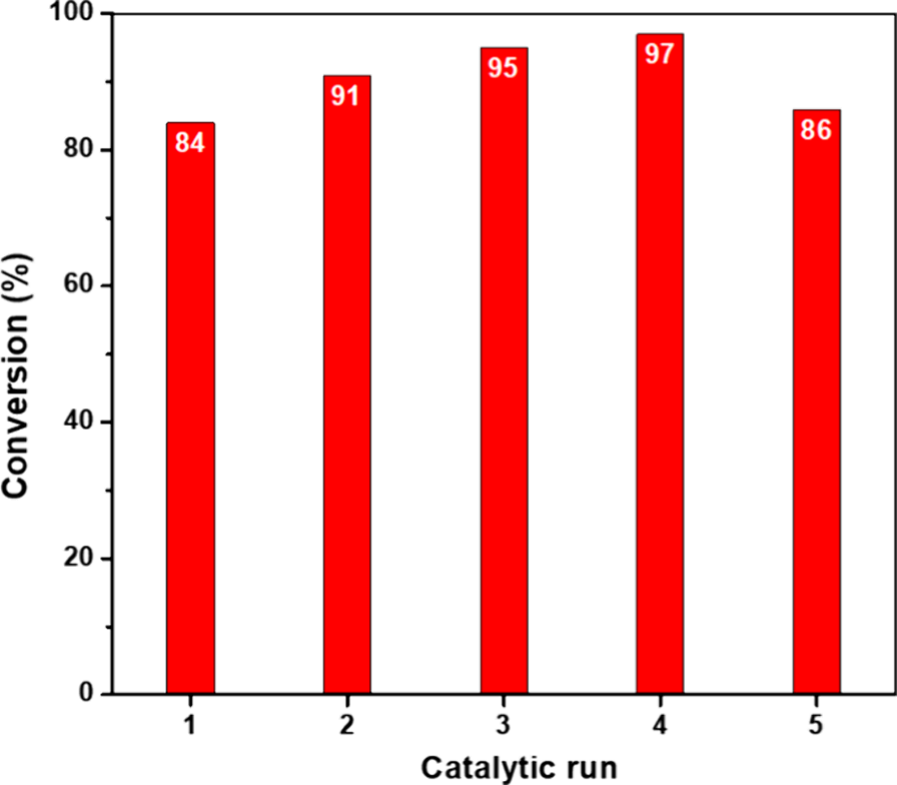
Recyclability tests for **QOF1-N** material in the photocatalytic oxidation of FA.

To better understand the photocatalytic mechanism involved in the oxidation of FA, the reaction was carried out in the presence of 1,4-diazabicyclo[2.2.2]­octane (DABCO), a well-known singlet oxygen quencher (Scheme 2).^39^ Our results reveal that the oxidation reactions were completely inhibited for both **QOF1** and **QOF1-N** in the presence of DABCO (see Figure S22). This observation provides strong experimental evidence suggesting that ^1^O_2,_ generated through an energy transfer mechanism, might be the active reactive oxygen species (ROS) responsible for FA oxidation. To further validate the distinct singlet oxygen generation capabilities of QOF1 and QOF1-N, we investigated the oxidation of α-terpinene (see Table S3 and Figures S24–S26). This molecule serves as a selective probe for singlet oxygen, and its conversion to ascaridole is recognized as a quantitative measure of singlet oxygen production.^40^ Interestingly, our experiments indicate that QOF1-N exhibits a singlet oxygen generation rate twice as high as that of QOF1.

Based on our observations and supported by previous studies,^31^ it is plausible that the photocatalytic transformation of FA into 5H5F proceeds via the formation of an endoperoxide intermediate, resulting from the reaction of FA with photogenerated ^1^O_2_ (Scheme 3). Further rearrangement involving C–C oxygen insertion leads to the formation of an alkoxyl transient compound. This intermediate then undergoes a deformylation step, yielding the final 5H5F product. In this mechanism, the rate-limiting step is the generation of singlet oxygen, which is significantly enhanced when **QOF1-N** is used as the photocatalyst.

**Scheme 3:**
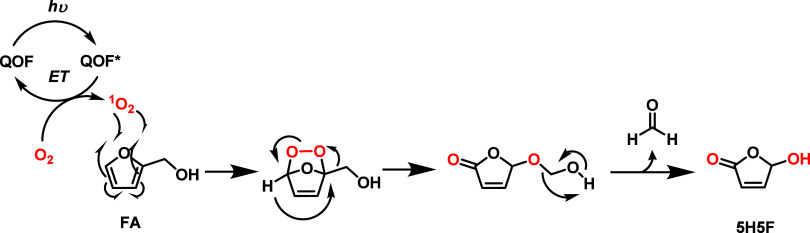
Proposed Reaction Pathway for the Formation of 5H5F Product

The recyclability of **QOF1-N** for the photooxidation of furfuryl alcohol was tested, concluding that the material preserved its photocatalytic activity for up to 5 catalytic runs (see Figure 5 and Figure S23).

The overall catalytic study reveals distinct photocatalytic behavior for **QOF1** and **QOF1-N**. **QOF1** demonstrates superior activity as a photocatalyst in a typical photoredox transformation, such as HER. In contrast, **QOF1-N** excels in energy transfer processes, particularly in generating singlet oxygen, which drives the selective oxidation of FA to 5H5F.

### Theoretical Calculations

Density functional theory (DFT) calculations (M06/6–31G­(d,p)) were performed to gain insights into the electronic structures of **QOF1** and **QOF1-N** (see Supporting Information for computational details). Four models of increasing size were considered (Figure S27), and the results indicated that the trends were not sensitive to the model size (Figures S28–S30). Therefore, we focused on the results obtained for the largest 6U model. In **QOF1**, the ring structure exhibits a slight deviation from planarity (Figure 6) due to repulsive interactions between the C–H groups of the benzene and quinoline fragments. This results in a mean dihedral angle of 14.6° between the rings (Figure 6). **QOF1-N** has two isomeric forms based on the relative positioning of the nitrogen atoms in the quinoline and pyridine fragments. In *E*-**QOF1-N**, the nitrogen atoms are located at opposite sides of the C—C bond connecting the quinoline and pyridine rings, whereas in *Z*-**QOF1-N**, both nitrogen atoms are on the same side. *E-* **QOF1-N** is planar, with a mean dihedral angle of 0.7° as it does not have opposing C—H groups from the quinoline and pyridine rings. In contrast, *Z-* **QOF1-N** experiences two destabilizing interactions between the quinoline and pyridine rings: the repulsion between two facing C—H groups and the repulsion between the lone pair electrons of the nitrogen atoms. This leads to a larger dihedral angle of 29.8°. Consequently, the ring planarity follows the order: *Z-* **QOF1-N** < **QOF1** < *E-* **QOF1-N**. The *E*-**QOF1-N** isomer is more stable than the *Z*-**QOF1-N** isomer by 43.2 kcal·mol^–1^ (approximately 7 kcal·mol^–1^ per quinoline unit), a trend that holds for most of the models considered. This increased stability of *E*-**QOF1-N** is attributed to the absence of repulsive interactions between the nitrogen atoms and the C—H groups. As a result, the electronic structure discussion herein will focus on *E*-**QOF1-N**, assuming it to be the most abundant isomer of **QOF1-N**.

**Figure 6:**
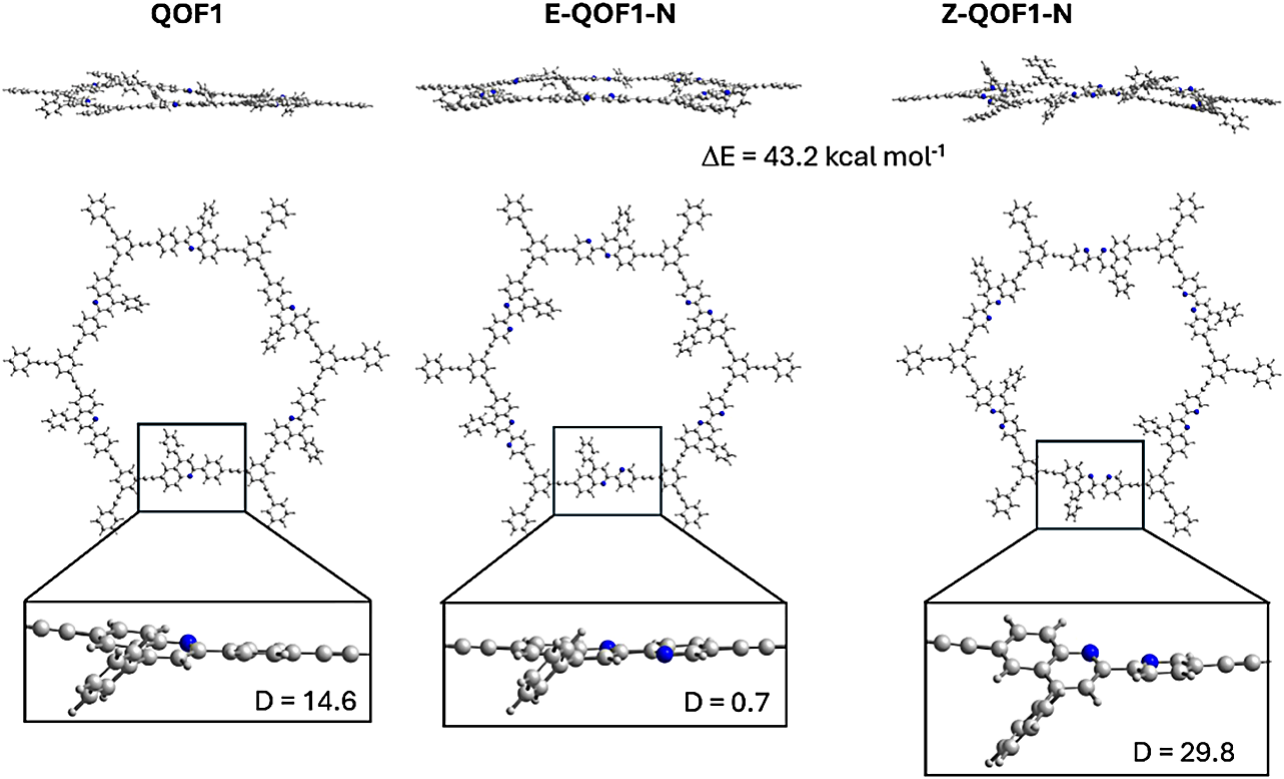
Optimized structures of **QOF1** and **QOF1-N** obtained with the 6U model.

Time-dependent (TD-) DFT calculations on the photochemical process reveal that the computed absorption wavelengths for **QOF1** and *E*-**QOF1-N** are strikingly similar (Figure 7). Both QOFs exhibit transitions involving π-molecular orbitals that are delocalized across the six quinoline-pyridine units and linking triple bonds. This is reflected in the nearly identical computed HOMO–LUMO energy gaps (3.73 eV for **QOF1** and 3.71 eV for *E*-**QOF1-N**), which align well with the experimental band gaps, despite the differences in absolute values between the experimental and theoretical results. Figure 7 shows the occupied and unoccupied orbital involved in the excitation transition of both materials. In each case, the occupied and unoccupied molecular orbitals are highly delocalized, with significant contributions from the quinoline, pyridine, and triple bond fragments. Notably, the molecular orbitals in *E*-**QOF1-N** exhibit greater delocalization than **QOF1**, reflecting the higher planarity of *E*-**QOF1-N**. In this isomer, contributions from the six quinoline-pyridine fragments are nearly equivalent, whereas in **QOF1**, they are not. This enhanced delocalization is particularly evident in the lowest unoccupied molecular orbitals (LUMO) of both materials. Importantly, despite negligible changes in the band gap, the presence of an additional nitrogen atom in **QOF1-N** compared to **QOF1** results in a stabilization of both the highest occupied molecular orbitals (HOMO) and the LUMO by 0.12 eV. This result is in good agreement with the experimental observations from the XPS measurements in the VB region for both **QOF1** and **QOF1-N** materials (*vide supra*). Stabilizing the LUMO of **QOF1-N** is expected to inhibit the material’s photooxidation, likely contributing to its reduced electron transfer capacity. This stabilization provides a plausible explanation for why **QOF1-N** is less efficient as a catalyst for HER. However, the increased conjugation in this N-enriched material may facilitate enhanced relaxation from its excited state, explaining its superior performance in energy transfer phenomena.

**Figure 7:**
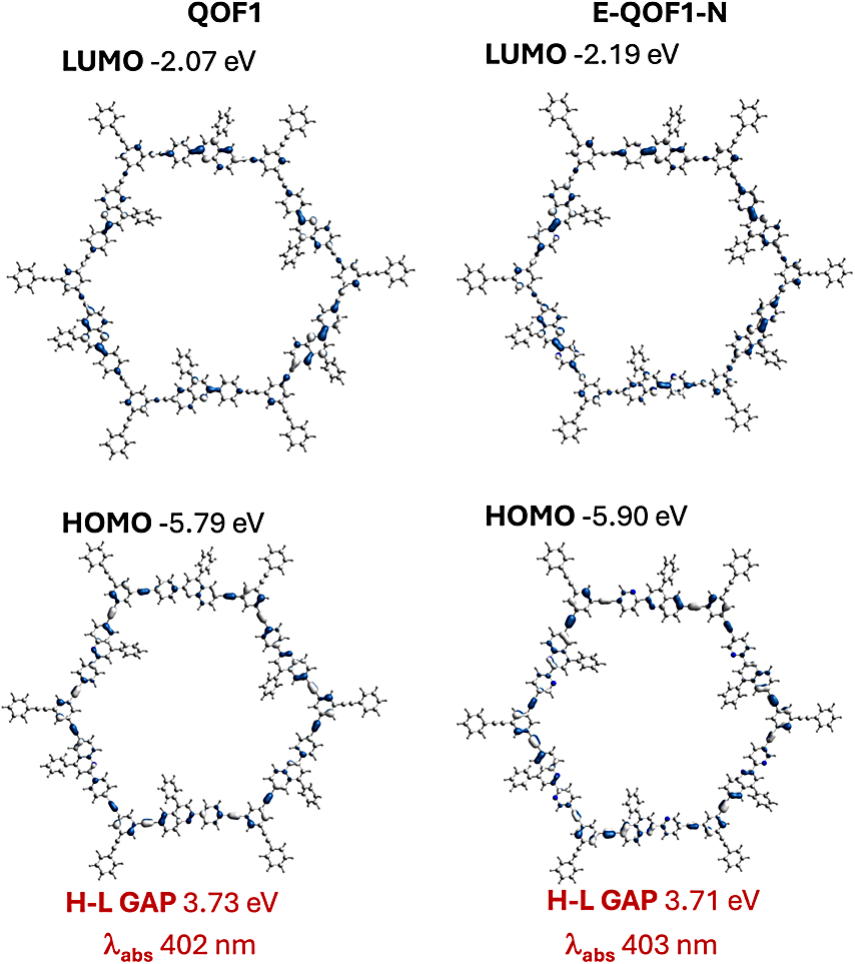
Molecular orbitals involved in the photochemical process of **QOF1** and **QOF1-N**.

## Conclusions

Two quinoline-containing CMPs have been successfully synthesized and characterized. The sole difference between these materials lies in substituting a phenyl fragment with a pyridine moiety in a position adjacent to the quinoline core. Despite their similar structural and photophysical properties, the divergent photocatalytic activities of **QOF1** and **QOF1-N** highlight the impact of nitrogen enrichment on their performance. The higher nitrogen content in **QOF1-N** enhances energy transfer processes from the excited photocatalyst to molecular oxygen, increasing ROS generation on shorter time scales. In contrast, **QOF1** demonstrates superior performance as a photocatalyst for HER, a process predominantly governed by electron transfer. Theoretical analyses indicate that the presence of additional nitrogen atoms in **QOF1-N**, due to their electronegativity, stabilizes both the valence and conduction bands, thereby reducing its efficiency for electron transfer from the excited state. However, the reduced steric hindrance in **QOF1-N** promotes greater planarity and improved π-conjugation, which likely enhances its energy transfer capability.

These results highlight the pivotal role of nitrogen in tuning the photocatalytic properties of organic materials. This approach presents a powerful strategy for the rational design of building blocks in heterogeneous organic photocatalysts, broadening their applicability across a spectrum of photocatalytic processes, from oxidation reactions to hydrogen generation.

## Experimental Section

### Synthesis of 6-Iodo-2-(4-iodophenyl)-4-phenylquinoline (**1a**)

Into a 50 mL Schlenk tube 4-iodobenzaldehyde (668 mg, 2.88 mmol, 1 equiv), 4-iodoaniline (631 mg, 2.88 mmol, 1 equiv), phenylacetylene (302 μL, 2.88 mmol, 1 equiv) and Sc­(OTf)_3_ (142 mg, 0.288 mmol) were added over 3 mL of Toluene as solvent. Then, the mixture was heated to 110 °C for 48h under air atmosphere. After the reaction was completed, the solvent was removed under vacuum, and the crude mixture was purified by column chromatography (CyHex:DCM gradient 70:30 to 50:50) to obtain quinoline **1a** as a light white solid (491 mg, 32% yield).

^1^H NMR (CDCl_3_, 300 MHz): δ 8.24 (d, J = 1.6 Hz, 1H), 7.97 (m, 2H), 7.94 (dd, J = 8.7, 1.6 Hz, 2H), 7.85 (dd, J = 8.7, 1.6 Hz, 2H), 7.77 (s, 1H), 7.61–7.50 (m, 5H).

^13^C NMR (CDCl_3_, 75 MHz): δ 156.1, 148.5, 147.7, 138.6, 138.1, 137.6, 134.5, 131.8, 129.5, 129.2, 128.9, 128.9, 127.7, 119.4, 96.4, 92.5.

### Synthesis of 6-Bromo-2-(5-bromopyridin-2-yl)-4-phenylquinoline (**1b**)

Into a 50 mL Schlenk tube 5-bromopinacolaldehyde (536 mg, 2.88 mmol, 1 equiv), 4-bromoaniline (495 mg, 2.88 mmol, 1 equiv), phenylacetylene (302 μL, 2.88 mmol, 1 equiv) and Sc­(OTf)_3_ (142 mg, 0.288 mmol) were added over 3 mL of Toluene as solvent. Then, the mixture was heated to 110 °C for 48h under air atmosphere. After the reaction was completed, the solvent was removed under vacuum, and the crude mixture was purified by column chromatography (CyHex:DCM gradient 70:30 to 50:50) to obtain quinoline **1b** as a light white solid (342 mg, 27% yield).

^1^H NMR (CDCl_3_, 300 MHz): δ 8.75 (dd, J = 2.3, 0.6 Hz, 1 H), 8.60 (dd, J = 8.5, 0.6 Hz, 1H), 8.50 (s, 1H), 8.09 (m, 2H), 8.03–7.99 (dd, J = 8.6, 2.3 Hz, 1H), 7.83–7.79 (dd, J = 8.6, 2.3 Hz, 1H), 7.57–7.55 (m, 5H).

^13^C NMR (CDCl_3_, 75 MHz): δ 155.0, 154.4, 150.2, 148.6, 147.1, 139.6, 137.6, 133.1, 131.9, 129.5, 128.8, 128.7, 128.0, 123.1, 121.9, 121.3, 119.7.

### Synthesis of **QOF1** Material

Into a 250 mL Schlenk tube, quinoline **1a** (850 mg, 1.59 mmol, 1.5 equiv) and 1,3,5-triethynylbenzene (159 mg, 1.06 mmol, 1 equiv) were added, followed by a 20% mol load of CuI (45.3 mg, 0.238 mmol) and 10% mol load of Pd­(PPh_3_)_4_ (123 mg, 0.119 mmol) respect to the quinoline. Then, the Schlenk was sealed with a septum, and 3 Ar-vacuum cycles were applied. After the cycles, and under Ar, the purged solvent consisting of a mixture of toluene and NEt_3_ (1:1) (30 mL) was added through the septum, and the mixture was heated up to 100 °C during 48h. After the reaction was completed, the resulting solid was filtered over a pad, and washed with warm MeOH, CHCl_3_ and acetone. After the washing, the dark-yellow solid was further cleaned to remove Pd residue from the synthesis, suspending it on a sodium diethyldithiocarbamate solution (0.01 g/mL) (50 mL) in methanol, heating the suspension to 75 °C overnight. Once the dark color of the powder was removed, the solid was filtrated again and washed further with MeOH and Acetone, drying it under vacuum to obtain the final **QOF1** and as an intense yellow fine powder (532 mg, 88% yield).

### Synthesis of **QOF1-N** Material

Into a 250 mL Schlenk tube, quinoline **1b** (400 mg, 0.9 mmol, 1.5 equiv) and 1,3,5-triethynylbenzene (91 mg, 0.6 mmol, 1 equiv) were added, followed by a 20% mol load of CuI (23.1 mg, 0.121 mmol) and 10% mol load of Pd­(PPh_3_)_4_ (70 mg, 0.06 mmol) respect to the quinoline. Then, the Schlenk was sealed with a septum, and 3 Ar-vacuum cycles were applied. After the cycles, under Ar, the purged solvent consisting of a mixture of toluene and NEt_3_ (1:1) (10 mL) was added through the septum, and the mixture was heated up to 100 °C for 48h. After the reaction was completed, the resulting solid was filtered over a pad and washed with warm MeOH, CHCl_3_, and acetone. After the washing, the dark-yellow solid was further cleaned to remove Pd residue from the synthesis, suspending it on a sodium diethyldithiocarbamate solution (0.01 g/mL) (25 mL) in methanol, heating the suspension to 75 °C overnight. Once the dark color of the powder was removed, the solid was filtrated again and washed further with MeOH and Acetone, drying it under vacuum to obtain the final **QOF1-N** and as an intense yellow fine powder (290 mg, 84% yield).

### General Procedure for HER Experiments

In a 22 mL glass vial, 13.4 mL of MeCN, 2.8 mL of NEt_3,_ 0.8 mL DI (deionized), and 50 μL of a solution of H_2_PtCl_6_·6 H_2_O (with a [Pt] = 294 ppm) were added. 2.5 mg of the QOF catalyst was then suspended in the mixture, resulting in a Pt w % of 5.5%. The sealed glass vial was subsequently purged with Ar for 10 min while sonicating to homogenize the suspension. The reaction vial was then irradiated with a 300 W Xe Lamp having a cutoff filter (>420 nm) from a distance of 10 cm for 4 h. After the reaction was quenched, 200 μL of the gaseous product was abstracted from the vial′s head space and analyzed using a Gas Chromatogram. The amount of H_2_ was calculated from integrating the peak area and interpolating from a lineal fit calibration.

For the recyclability experiments, the vial with the crude mixture was purged for 10 min with Ar to remove all the H_2_ produced during the previous cycle, before the subsequent catalytic runs were consecutively performed for 2 h irradiation.

### General Procedure for Furfuryl Alcohol Photooxidation Experiments

In a 7 mL glass vial, 3 mL of a 10 mM furfuryl alcohol (FA) solution in MeCN was added, then, 2.5 mg of the QOF catalyst was suspended. The mixture was purged with O_2_ for 10 min and irradiated using a 300 W Xe Lamp with a cutoff filer (>420 nm) from a distance of 10 cm during the corresponding reaction times. Quenching experiments with DABCO were carried out following the general procedure and adding 3 equiv of the quencher (with respect to the FA, 0.09 mmol) to the mixture.

For the recyclability experiments, the material was isolated after each catalytic cycle by centrifugation (4000 rpm) and washed with MeCN and acetone before being tested in the next cycle. Small losses in the catalyst load were compensated with fresh material to fill up to 2.5 mg for each cycle. The conversions to 5-hydroxy-2­(5H)-furanone were measured by peak integration from ^1^H NMR crude spectra.

For α-terpinene photocatalytic oxidation experiments, we followed the general procedure established for furfuryl alcohol oxidation, substituting 3 mL (0.3 mmol) of a 100 mM α-terpinene solution as the substrate. The reaction was irradiated for 45 min, with aliquots taken at 15 min intervals. The crude reaction mixtures were analyzed by ^1^H NMR, and product conversion to ascaridole was quantified through peak integration.

## Supplementary Material



## Notes

Additional experimental details and characterization, including FTIR, UV–vis, TGA, BET, and SEM/EDX (PDF)
