# Supplementary material for: Quinoline-Based Organic Frameworks with Divergent Photocatalytic Properties for Hydrogen Production and Furfuryl Alcohol Oxidation
Source: ACS Appl Eng Mater. 2025 Mar 28;3(4):927–37. doi: 10.1021/acsaenm.5c00044 (PMC12131186; doi:10.1021/acsaenm.5c00044)
Supplement: Supplementary file 1 [file em5c00044_si_001.pdf]

SUPPORTING INFORMATION

Quinoline-based Organic Frameworks with Divergent Photocatalytic Properties for Hydrogen Production and Furfuryl Alcohol Oxidation

Miguel Sánchez-Fuente,± Emmanuel N. Musa,√ Ankit K. Yadav, √ Kyle T. Smith, √ Christopher N. Young,† Alicia Moya,± Xavier Solans-Monfort,§ Kyriakos C. Stylianou√, *, Rubén Mas-Ballesté±,¥*

± Department of Inorganic Chemistry (Module 7), Facultad de Ciencias, Universidad Autónoma de Madrid, 28049 Madrid, Spain.

§ Department of Chemistry (Module 7), Facultat de Ciencies, Universitat Autònoma de Barcelona, 08191 Bellaterra, Spain.

√ Materials Discovery Lab (MaD Lab), Department of Chemistry, Oregon State University, Corvallis, Oregon 97331, United States.

† Analytical and Development Labs, HP Inc., 1000 NE Circle Blvd., Corvallis, Oregon, 97330, United States

¥ Institute for Advanced Research in Chemical Sciences (IAdChem), Universidad Autónoma de Madrid, 28049 Madrid, Spain.

Email. kyriakos.stylianou@oregonstate.edu; ruben.mas@uam.es

Contents

1. General Materials and Methods.
2. Computational details
3. Synthesis of the quinoline building units.
   3.1 Synthesis of 6-iodo-2-(4-iodophenyl)-4-phenylquinoline (**1a**).
   3.2 Synthesis of 6-bromo-2-(5-bromopyridin-2-yl)-4-phenylquinoline (**1b**).
4. Synthesis of QOF materials.
5. Comparative FTIR and Solid-state CP/MAS ^{13}C-NMR spectra of QOF1 and QOF1-N
6. Comparative full FTIR spectra of 1,3,5-triethynylbenzene, QOFs and their respective building units (1a-b)
7. XPS analysis of the materials
8. Kubelka Munk calculations for the optical band gap of the materials
9. Thermogravimetric analysis of the materials
10. SEM analysis of the materials as made
11. Nitrogen and CO_2 Isotherm analysis of the materials
12. Optimization of the catalyst loading and reaction time for HER experiments
13. Photocatalytic HER mechanism
14. EDX and SEM analysis of QOF1 after HER catalysis
15. XPS after HER catalysis
16. Optimization of the reaction time and catalytic load for FA oxidation experiments
17. ^{1}H-NMR of commercial 5H5F product 10mM solution in MeCN
18. ^{1}H-NMR spectra of FA oxidation experiments
19. α-terpinene oxidation experiments
20. Computational models
21. References

1. General Materials and Methods

Materials and instrumentation: All the starting reagents and solvents were purchased from BLD Pharm, Sigma Aldrich, or TCI.

Thermogravimetry Analysis (TGA)

Thermogravimetry Analysis (TGA) was carried out with a Thermogravimetry/Differential Thermal Analysis (TG-DTA) analyzer within the temperature range of 25–700°C with a heating rate of 10 °C/min under Ar flow (100 mL/min).

Scanning electron microscopy (SEM)

Scanning electron microscopy (SEM) images for the prepared catalysts were collected on a Quanta 600 3D, incorporated with an X-ray energy Dispersive Spectrometer (EDS).

Nitrogen and CO_2 adsorption-desorption isotherms

Nitrogen and CO_2 adsorption-desorption isotherm measurements were collected at 77K and 298K, respectively, using an IGA001 and ASAP2020 surface area analyzer. Before the measurement, the sample was degassed at 130 °C for 12 hr before data acquisition.

Fourier-Transform InfraRed (FTIR) spectroscopy

FT-IR spectra were recorded in a Perkin-Elmer 283 using KBr as a matrix for the pellet samples.

Characterization of oxidation products

Nuclear magnetic resonance (NMR) spectra were acquired on a Bruker AV-300 spectrometer (Bruker Corporation, Billerica, MA, USA), running at 300 MHz for ^{1}H and 75 MHz for ^{13}C. Chemical shifts (δ) are reported in ppm relative to residual solvent signals ($CDCl_3$: 7.26 ppm for ^{1}H-NMR, 77.0 ppm for ^{13}C-NMR). ^{13}C solid-state nuclear magnetic resonance was acquired on a Bruker AV-400 spectrometer coupled to a multinuclear probe ($^{15}N^{31}P$) CPMAS with triple channel (BL4 X/Y/1H) for a 4 mm rotor at room temperature, using 1k scans and 12 kHz of turning speed. The ^{1}H excitation pulse used is $\pi/2 \times 2.75$ µs, and the contact pulse is 3 ms.

X-ray photoelectron spectroscopy (XPS) studies

X-ray photoelectron spectroscopy (XPS) measurements were carried out under ultra-high vacuum conditions (10^{-10} mbar) using a SPECS GmbH electron spectroscopy system provided with a PHOIBOS 150 9MCD analyzer and a double anode X-ray source (Al/Mg). The samples were prepared as pellets mounted over a metallic substrate.

Diffuse reflectance (DR) UV-VIS spectroscopy

Diffuse reflectance (DR) UV-VIS spectra were obtained on a PerkinElmer UV-Vis Spectrometer by depositing the powders within a quartz slides window. The spectra were recorded at room temperature in the range of 200–800 nm, and the Kubelka-Munk function was applied to the raw data to eliminate scattering.

Photoluminescence (PL) studies

The photoluminescence (PL) emission QuantaMaster 8075-21 and a Horiba DeltaDiode 312 nm LED laser using time-correlated single photon counting (TCSPC). The PL spectra were collected from 0.333 mg/mL materials´ suspensions in ACN, at an excitation wavelength of 385 nm.

Scanning electron microscopy (SEM)/EDX The SEM images and energy-dispersive X-ray maps for some of the prepared catalysts were collected on a Quanta 600 3D, incorporated with an X-ray energy Dispersive Spectrometer (EDS).

2. Computational details

The computational strategy is similar to that we have recently successfully used to model other organic materials[1]. All calculations were performed with the Gaussian16 (Revision C.02) package[2]. The porous materials were represented by four finite models of increasing size (*Figure ST1*) to ensure that the primary photophysical behavior is not model dependent. Despite small differences between the models (*Figure T1, ST2-ST4*), the same trends were obtained when comparing the two porous materials, thus giving us further support that considering larger models would not influence the conclusions drawn.

All geometry optimizations were performed with the M06[3] DFT method and represented all atoms with the 6-31G(d,p)[4-6] double-z plus polarization basis sets. In addition, time-dependent DFT (TD-DFT) calculations at the optimized geometries were performed with the same level of theory to analyze the orbitals involved in the most favorable transitions. In these calculations, the lowest 20 singlet states were computed, although the most favorable transition in all cases involved most of the lowest energy transitions.

3. Synthesis of the quinoline building units

3.1 *Synthesis of 6-iodo-2-(4-iodophenyl)-4-phenylquinoline (**1a**)*

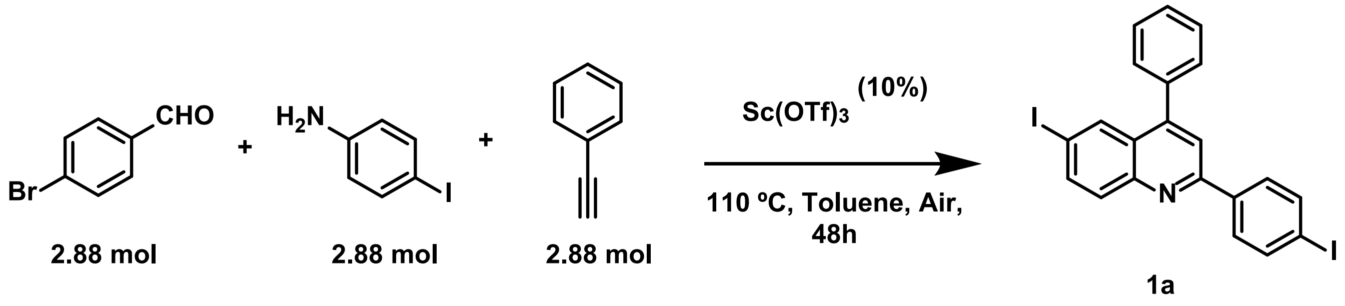

*Scheme S1: Synthesis of quinoline **1a***

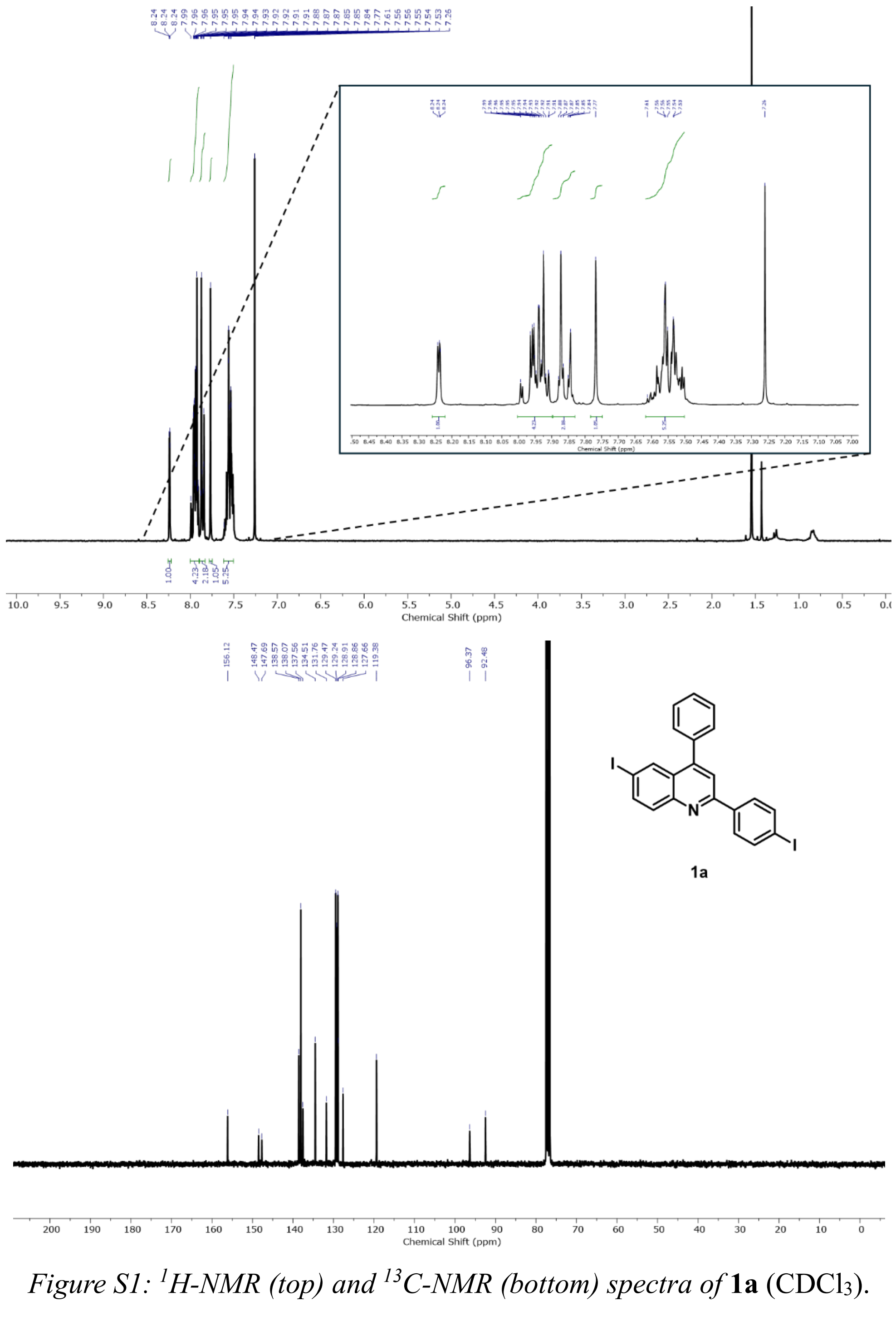

Figure S1: ^{1}H-NMR (top) and ^{13}C-NMR (bottom) spectra of **1a** ($CDCl_3$).

3.2 *Synthesis of 6-bromo-2-(5-bromopyridin-2-yl)-4-phenylquinoline* (**1b**)

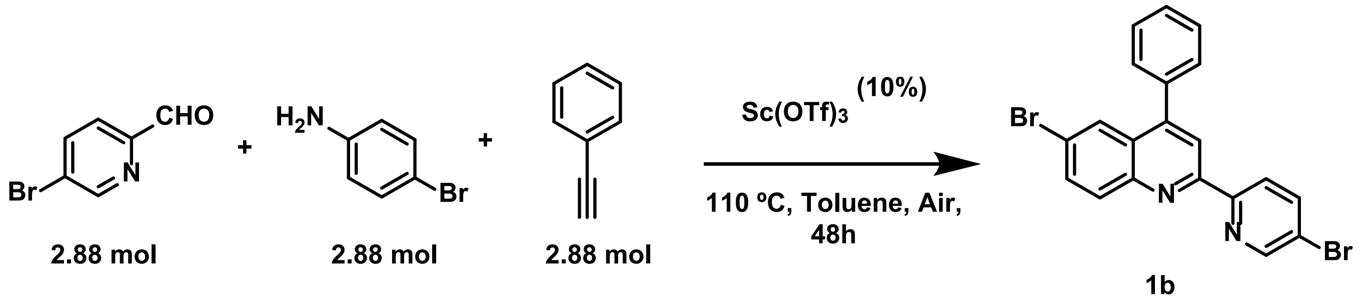

Scheme S2: Synthesis of quinoline ***1b***

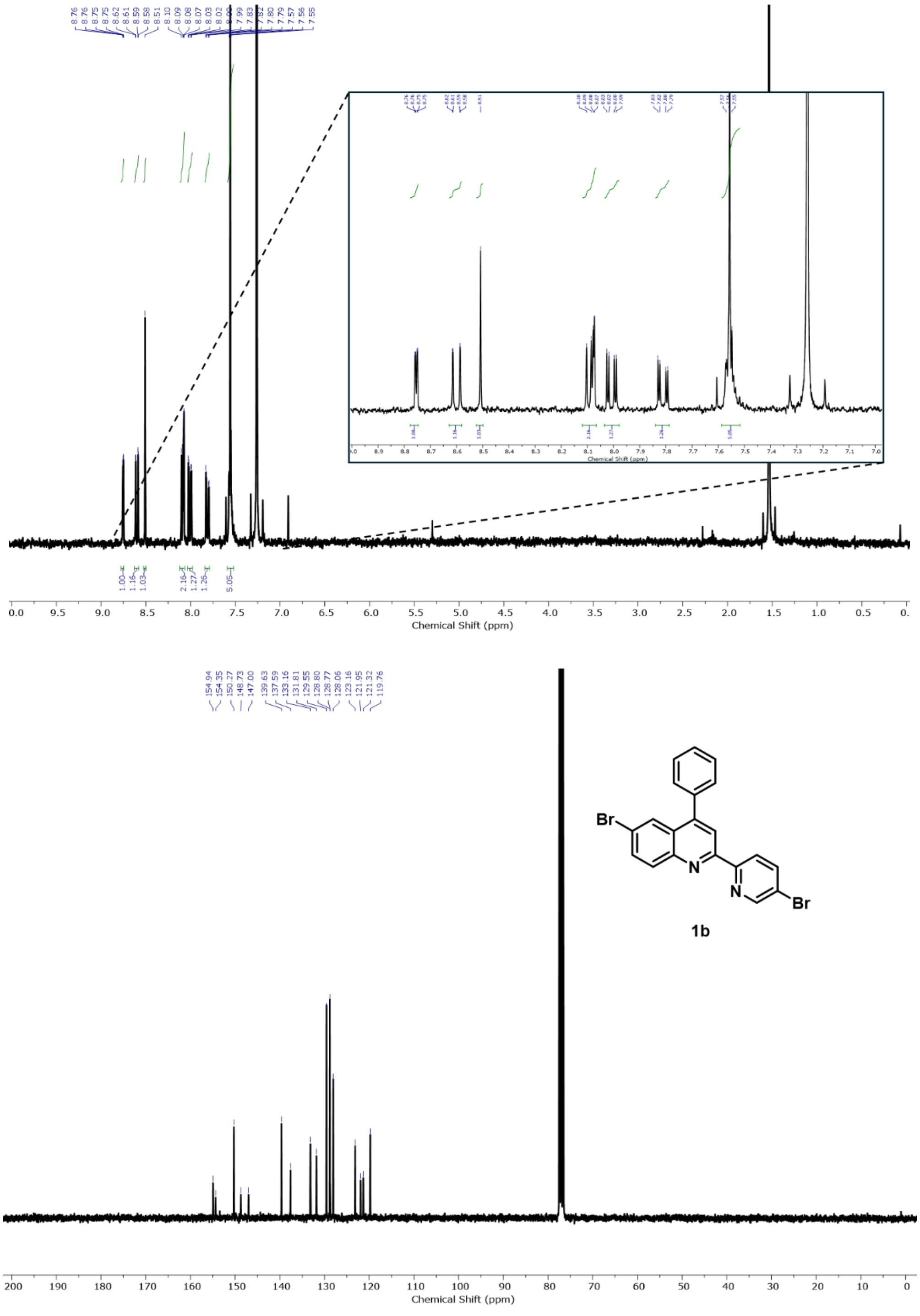

Figure S2: ^{1}H-NMR (top) and ^{13}C-NMR (bottom) spectra of **1b** ($CDCl_3$).

4. Synthesis of QOF materials

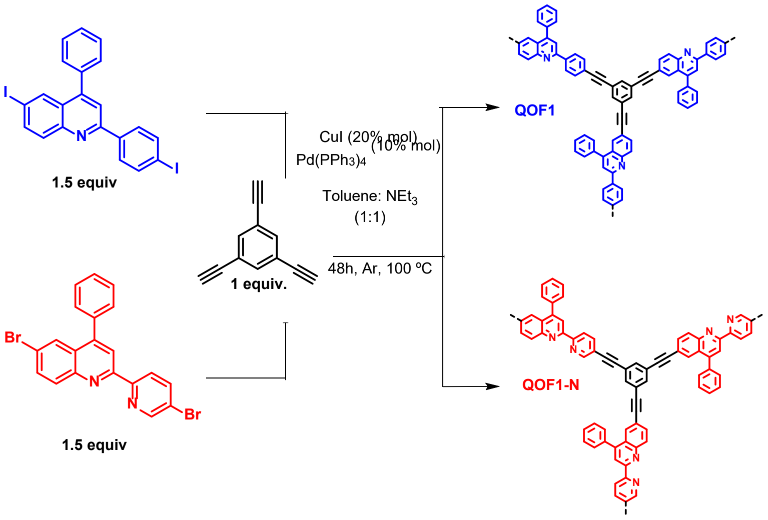

Scheme S3: Synthesis of QOF materials.

Table S1: Synthetic conditions for QOF1 and QOF1-N materials.

**Material**	**Quinoline**	**1,3,5-triethynylbenzene**	**CuI**	**Pd(PPh$_3$)$_4$**	**Solvent volume**	**NaS$_2$CN(C$_2$H$_5$)$_2$ Sol.**	**QOF amount**
QOF1	**1a** 850 mg (1.59 mmol)	159 mg (1.06 mmol)	45.3 mg	123 mg	30 mL	50 mL	532 mg (88% yield)
QOF1-N	**1b** 400 mg (0.9 mmol)	91 mg (0.6 mmol)	23.1 mg	70 mg	10 mL	25 mL	290 mg (84% yield)

5. Comparative FTIR and solid state CP/MAS ^{13}C-NMR of QOF1 and QOF1-N

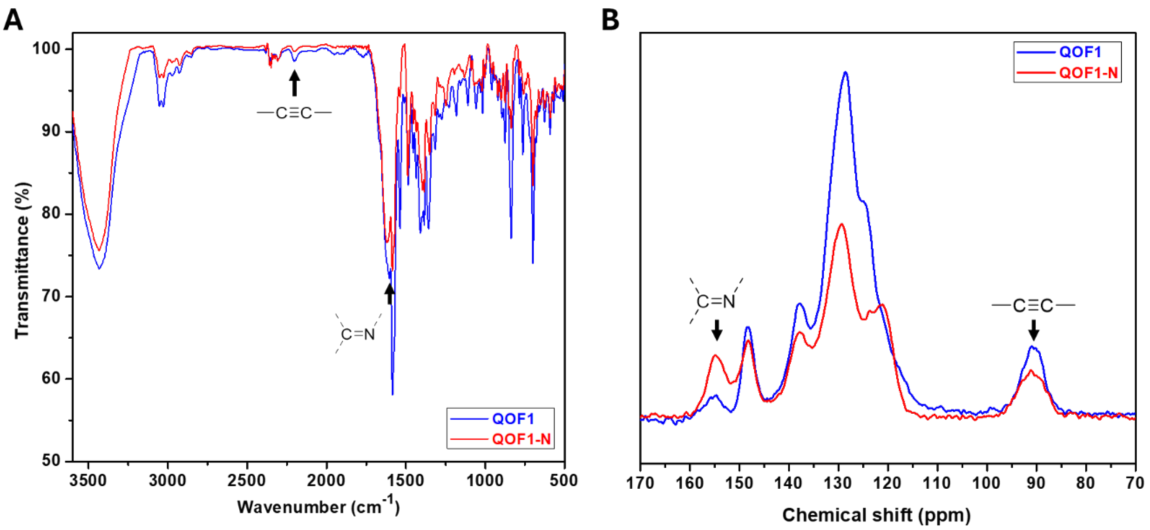

Figure S3: A) FTIR spectra in KBr and B) Solid state CP/MAS-^{13}CNMR spectra of QOF1 (blue) and QOF1-N (red).

6. Comparative full FTIR spectra of 1,3,5-triethynylbenzene, QOFs and their respective building units (1a-b)

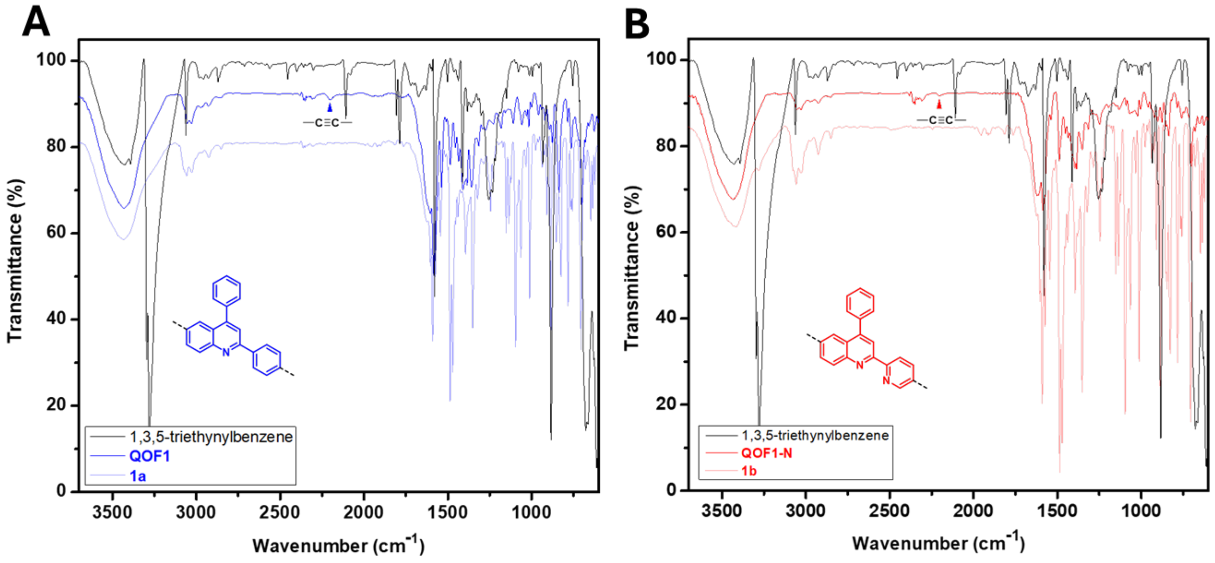

Figure S4: full FTIR spectra in KBr of A) 1,3,5-triethynylbenzene, QOF1 and 1a, B) 1,3,5-triethynylbenzene, QOF1-N and 1b

7. XPS analysis of the materials

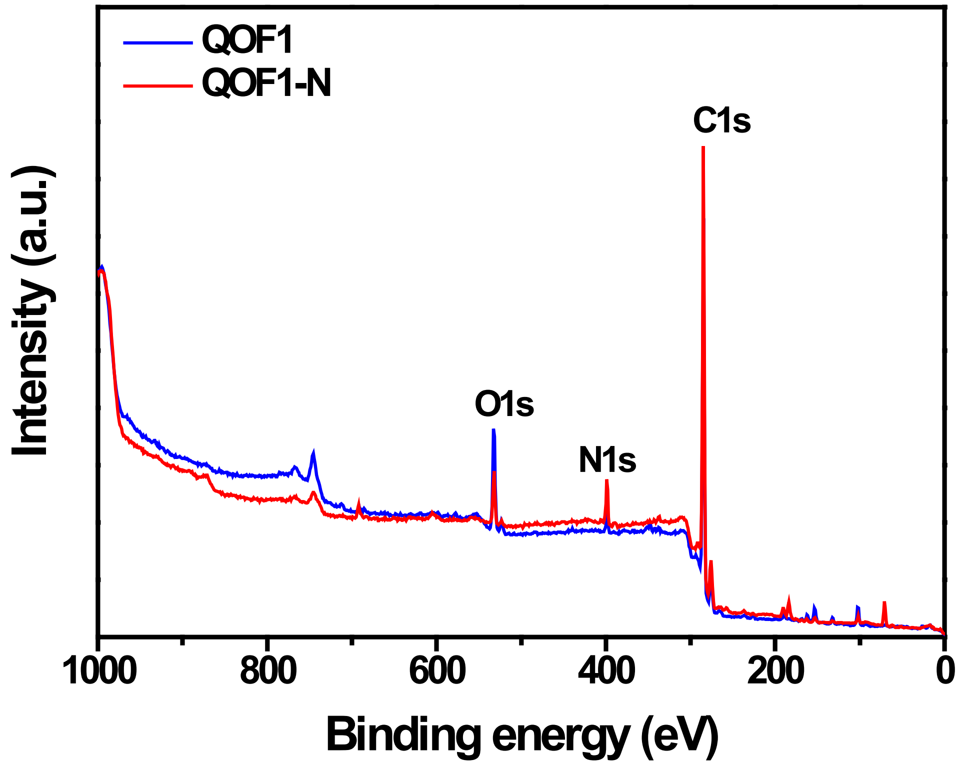

Figure S5: Survey XPS spectra of QOF1 (blue) and QOF1-N (red).

8. Kubelka Munk calculations for the optical band gap of the materials

The band gaps of the photocatalysts were estimated using the function (F(R)*hυ)λ vs. energy (eV), where λ is 2 for direct band gap, (F(R) is the Kubelka Munk function, h, is Planck constant, and υ is light frequency. The band gap values were obtained from the ratio between the intercept and the slope (a/b) from the linear fit (y=a+bx) obtained from the representation obtained.

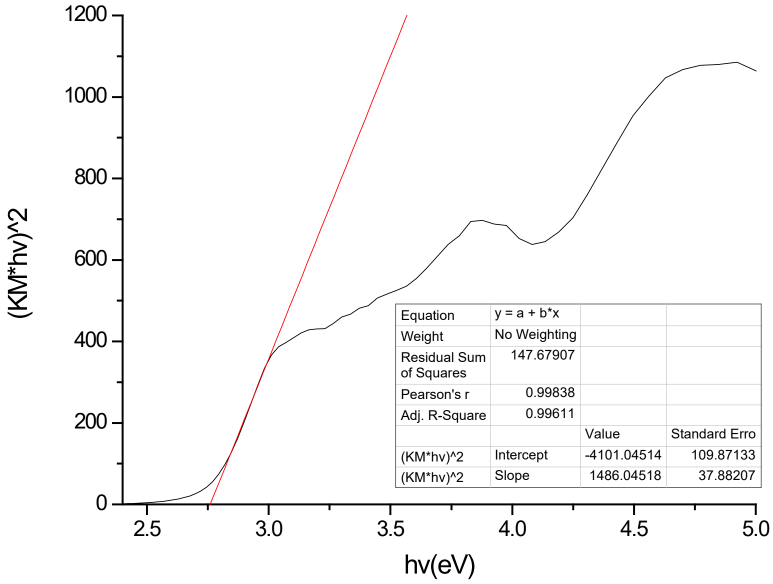

*Figure S6: Linear fit for the K-M direct band gap calculation for **QOF1**.*

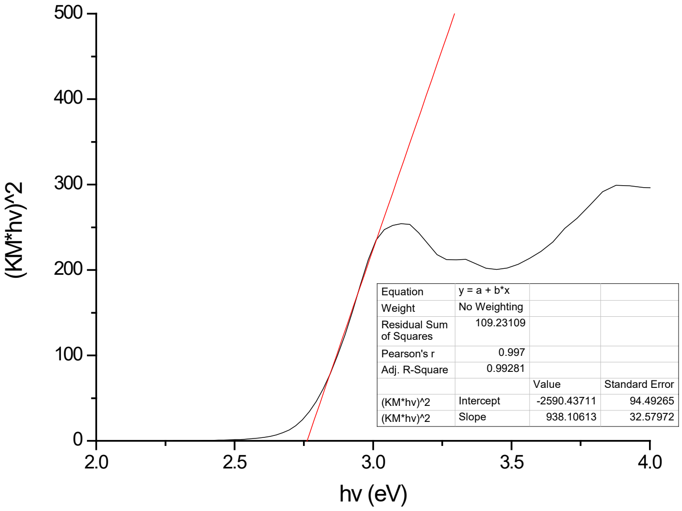

*Figure S7: Linear fit for the K-M direct band gap calculation for **QOF1-N**.*

Table S2: Direct bandgap for the photocatalytic materials.

**Material**	**Direct Band Gap (eV)**
QOF1	**2.76**
QOF1-N	**2.76**

9. Thermogravimetric analysis of the materials

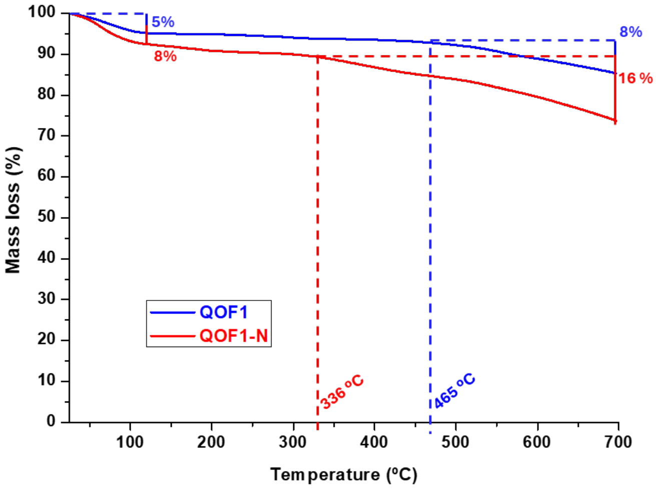

Figure S8: Thermogravimetric analysis of QOF1 (blue) and QOF1-N (red).

10. SEM analysis of the materials

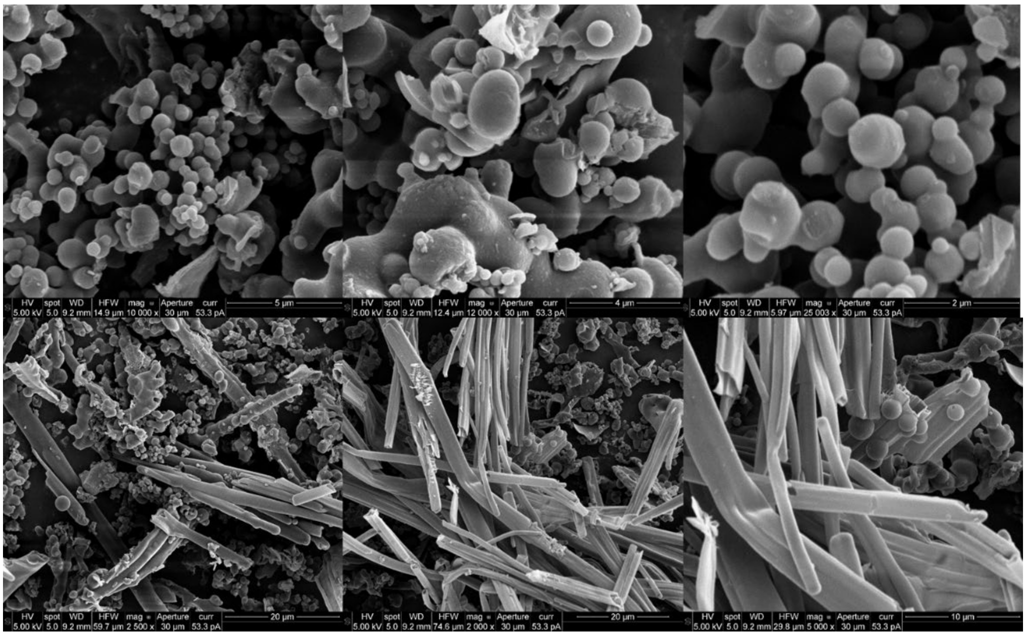

Figure S9: SEM images of QOF1 material.

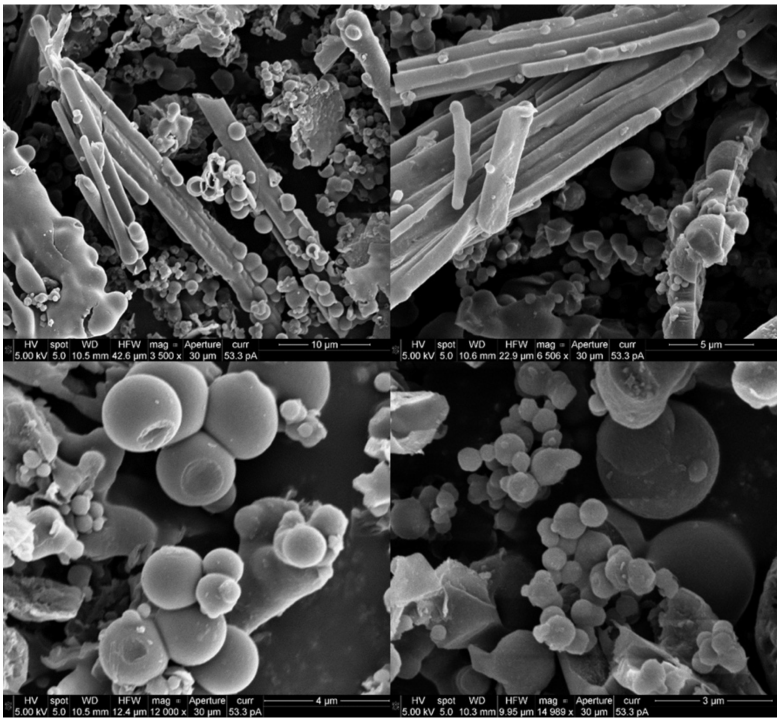

Figure S10: SEM images of QOF1-N.

11. Nitrogen and CO_2 Isotherm analysis of the materials

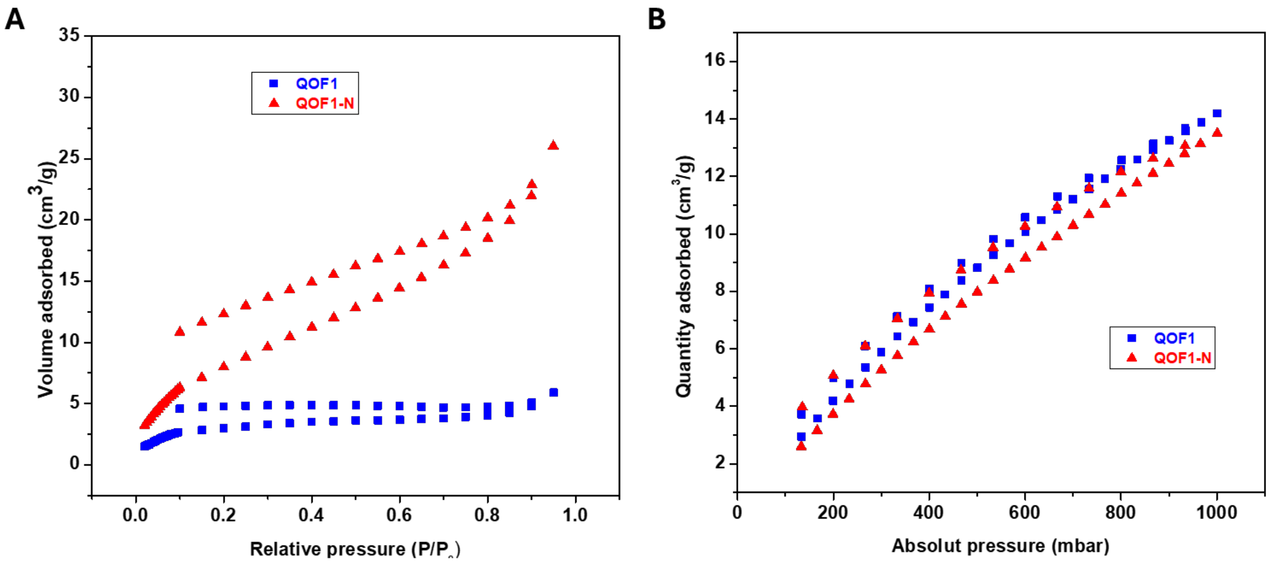

Figure S11: A) Nitrogen Adsorption/Desorption isotherms for QOF1 (blue) and QOF1-N (red). B) CO_2 Adsorption/Desorption isotherms for QOF1 (blue) and QOF1-N (red).

12. Optimization of the catalyst loading and reaction time for HER experiments

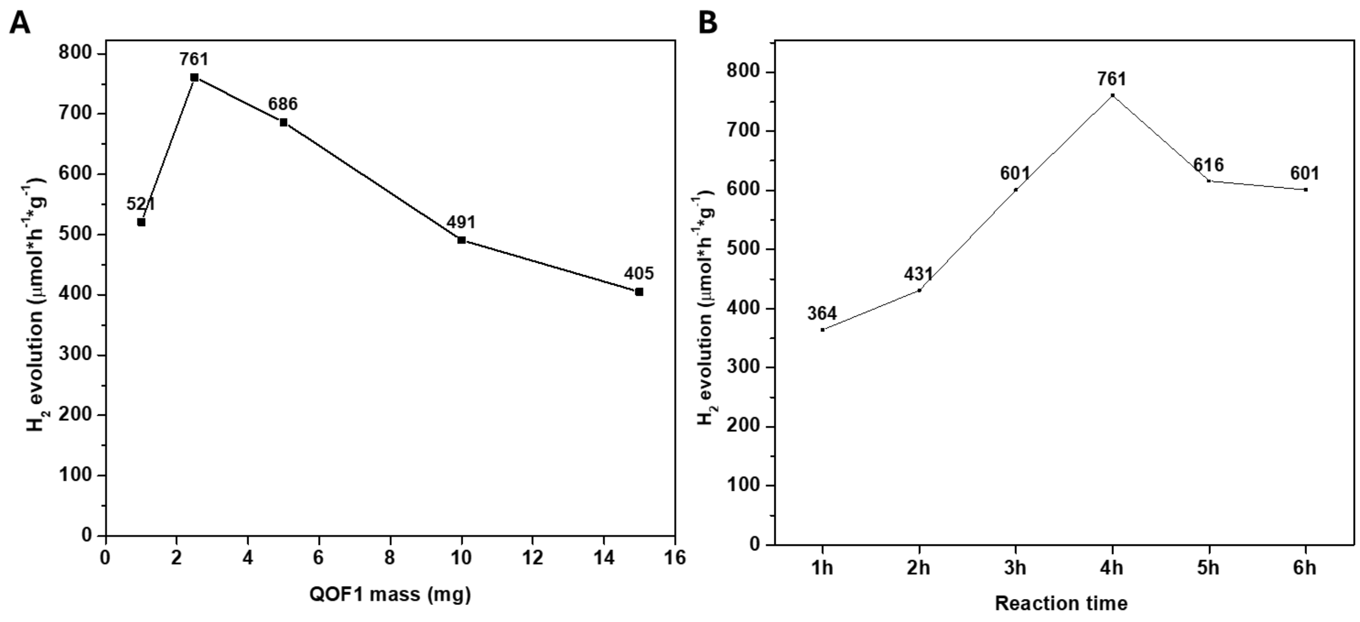

Figure S12: A) Optimization of the QOF1 catalyst loading for 4 h reaction time in presence of Pt (0.147 mg). B) HER rates with 2.5 mg of QOF1 catalyst loading for different reaction times in presence of Pt (0.147 mg, 5.5 wt%).

13. Photocatalytic HER mechanism

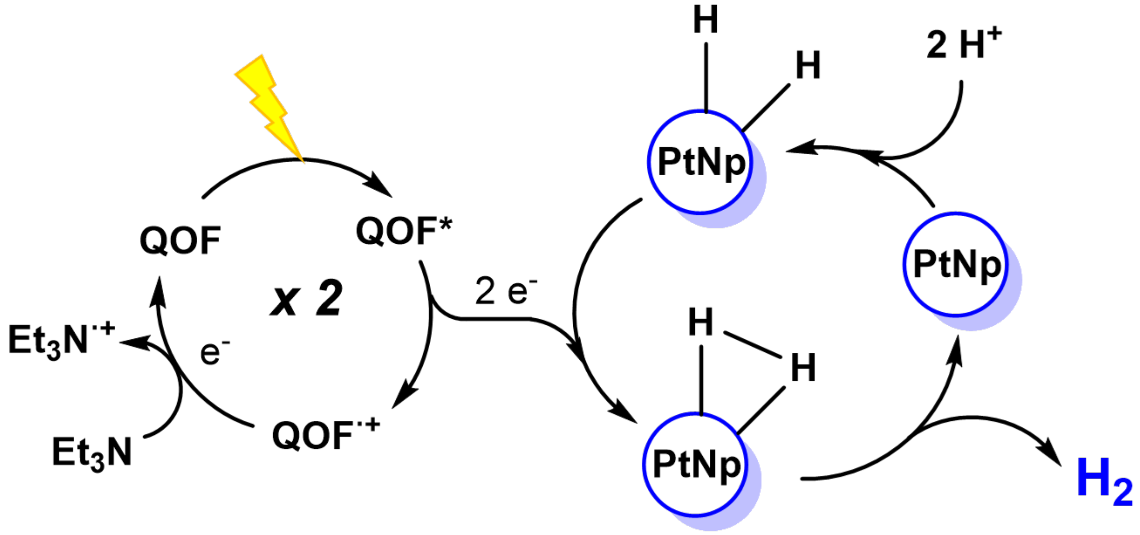

Scheme S4: Plausible mechanism for the photocatalytic HER system.

14. EDX, SEM and ATR-FTIR analysis of QOF1 after HER catalysis.

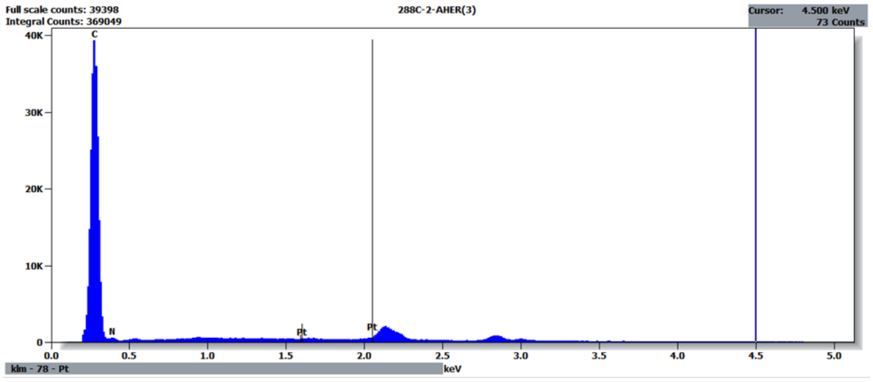

Figure S13: EDX analysis of the SEM-analyzed region of QOF1 catalyst.

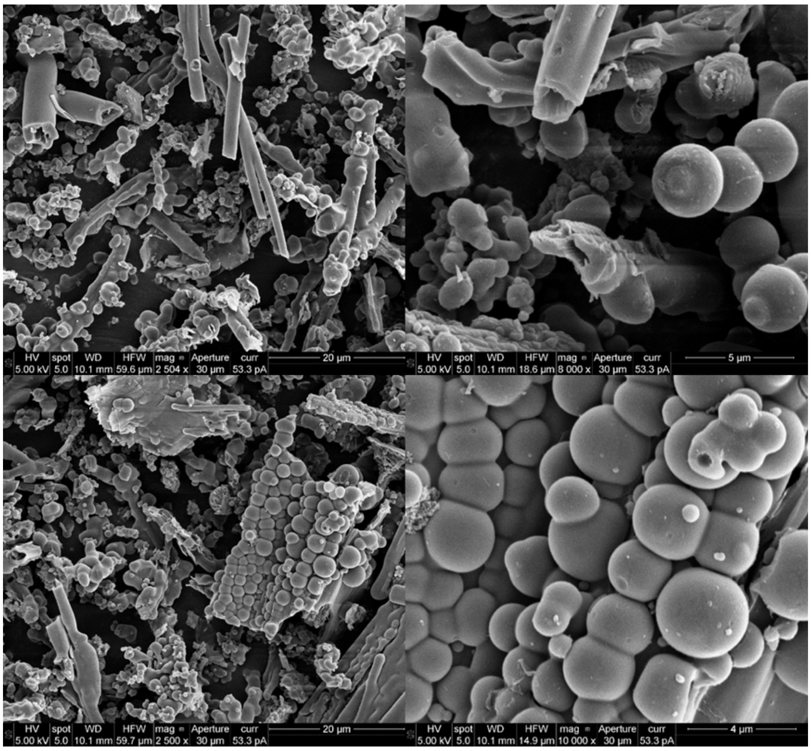

Figure S14: SEM images of QOF1 material after HER catalysis.

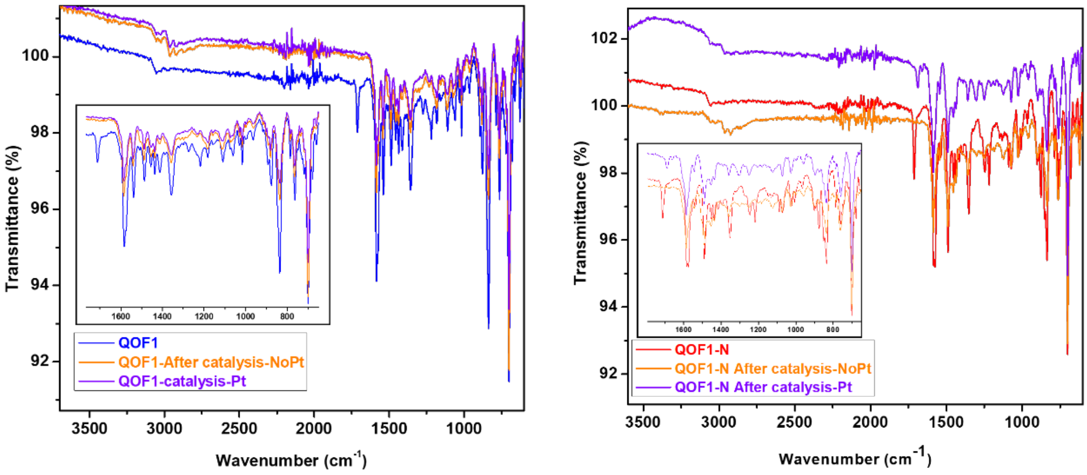

Figure S15: ATR-FTIR spectra of QOF1 and QOF1-N materials before and after HER catalysis (with and without Pt).

15. XPS analysis of QOF1 after HER catalysis

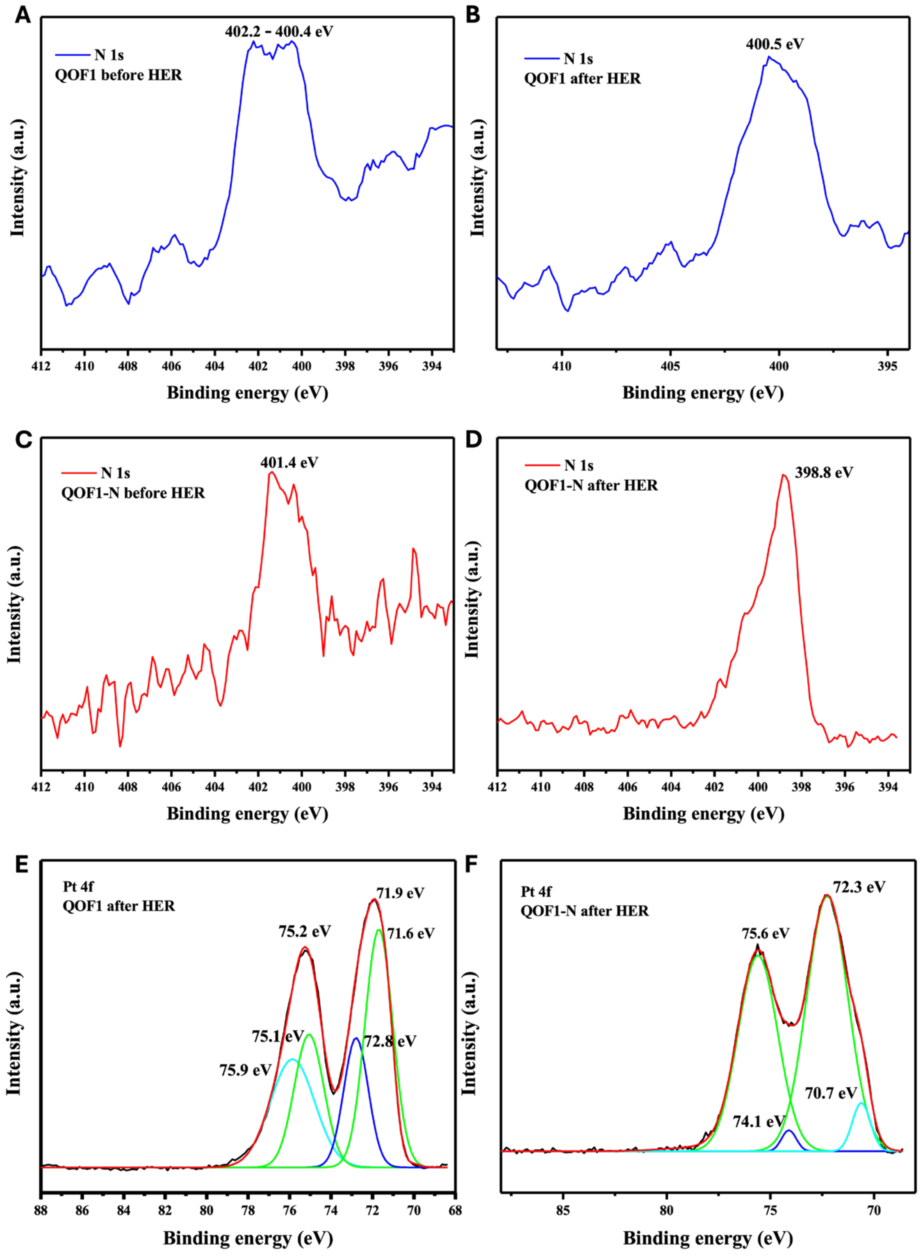

Figure S16: XPS analysis of QOF materials in presence of Pt cocatalyst: N1s for QOF1 A) before and B) after HER catalysis; N1s for QOF1-N C) before and D) after HER catalysis; and Pt4f for E) QOF1 and F) QOF1-N after HER catalysis.[7, 8]

16. Optimization of the reaction time and catalytic load for FA oxidation experiments

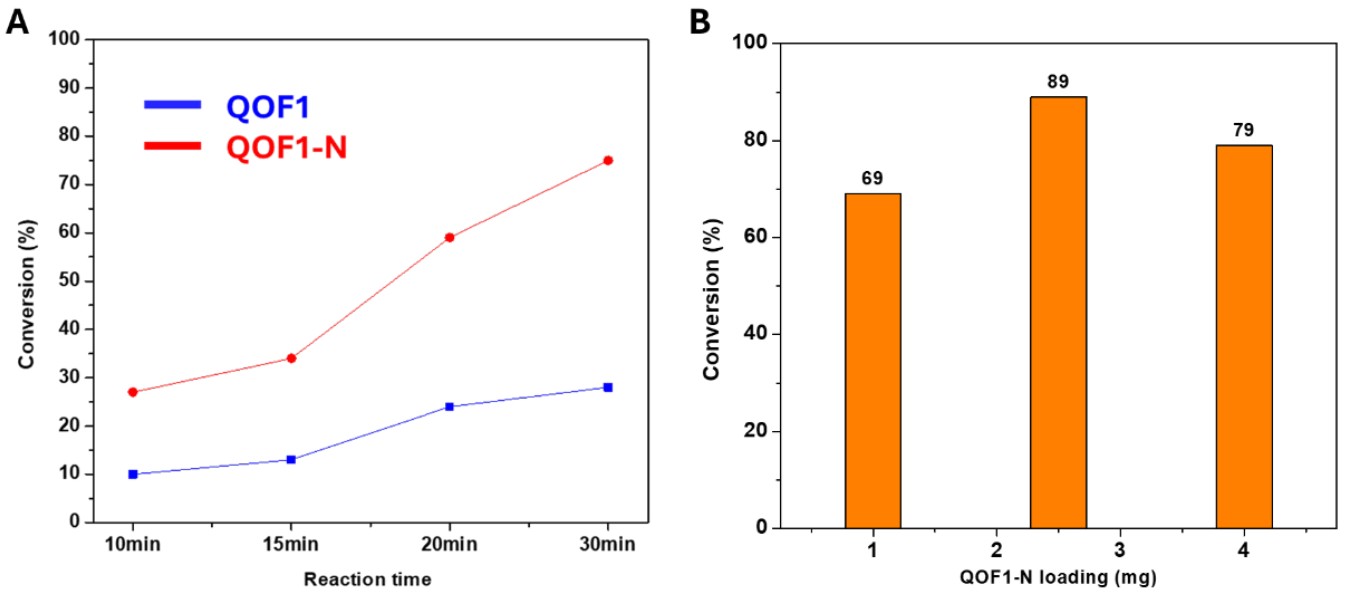

Figure S17: A) Conversion of FA photocatalytic oxidation reached with QOF1 (blue) and QOF1-N (red) for different reaction times. B) Optimization of QOF1-N catalyst loading for 30 min reaction time.

17. ^{1}H-NMR of commercial 5H5F product 10mM solution in MeCN

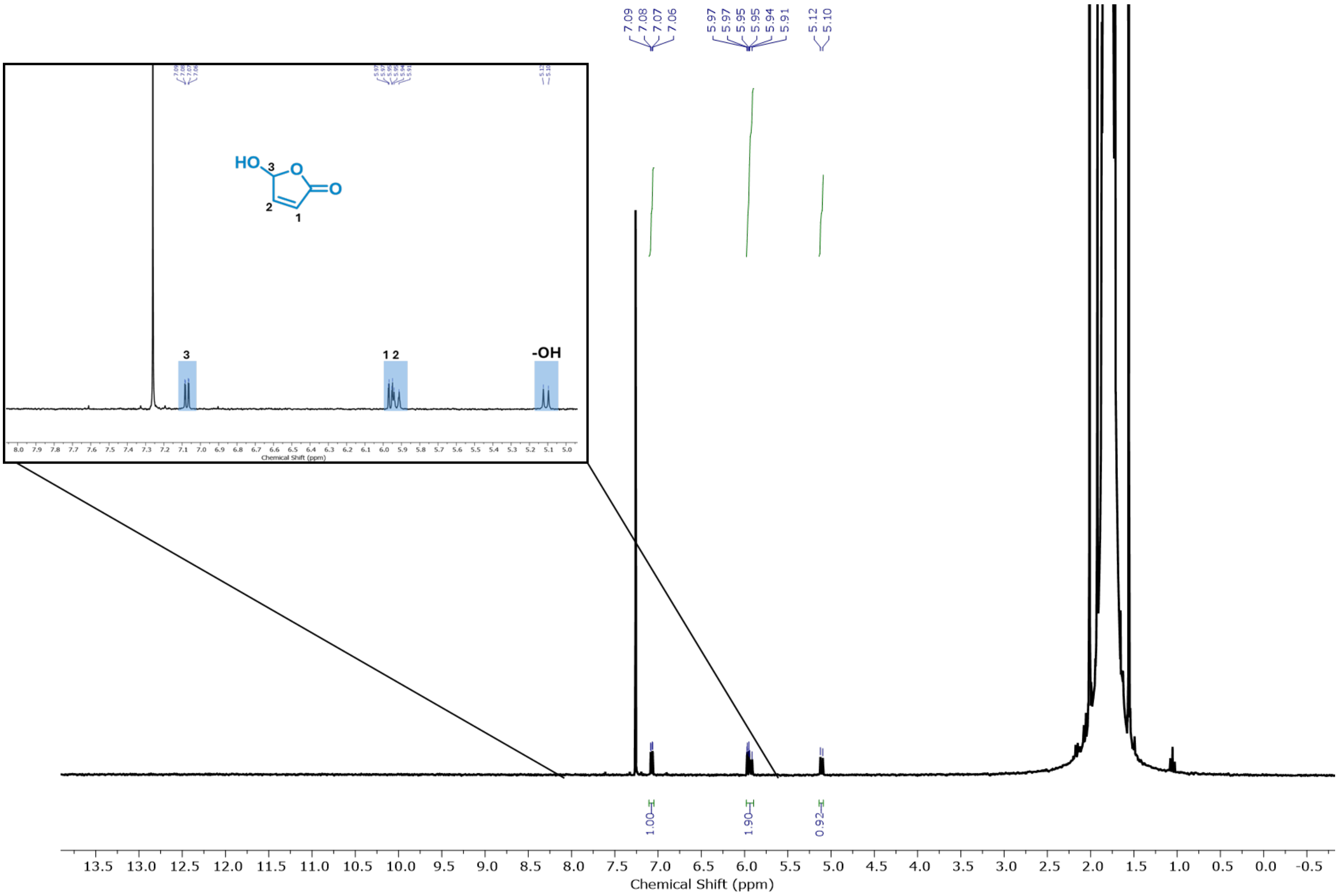

Figure S18: ^{1}H-NMR (300 MHz, $CDCl_3$) of a 10mM solution of commercial-sourced 5H5F product in MeCN.

18. ^{1}HNMR spectra of FA oxidation experiments

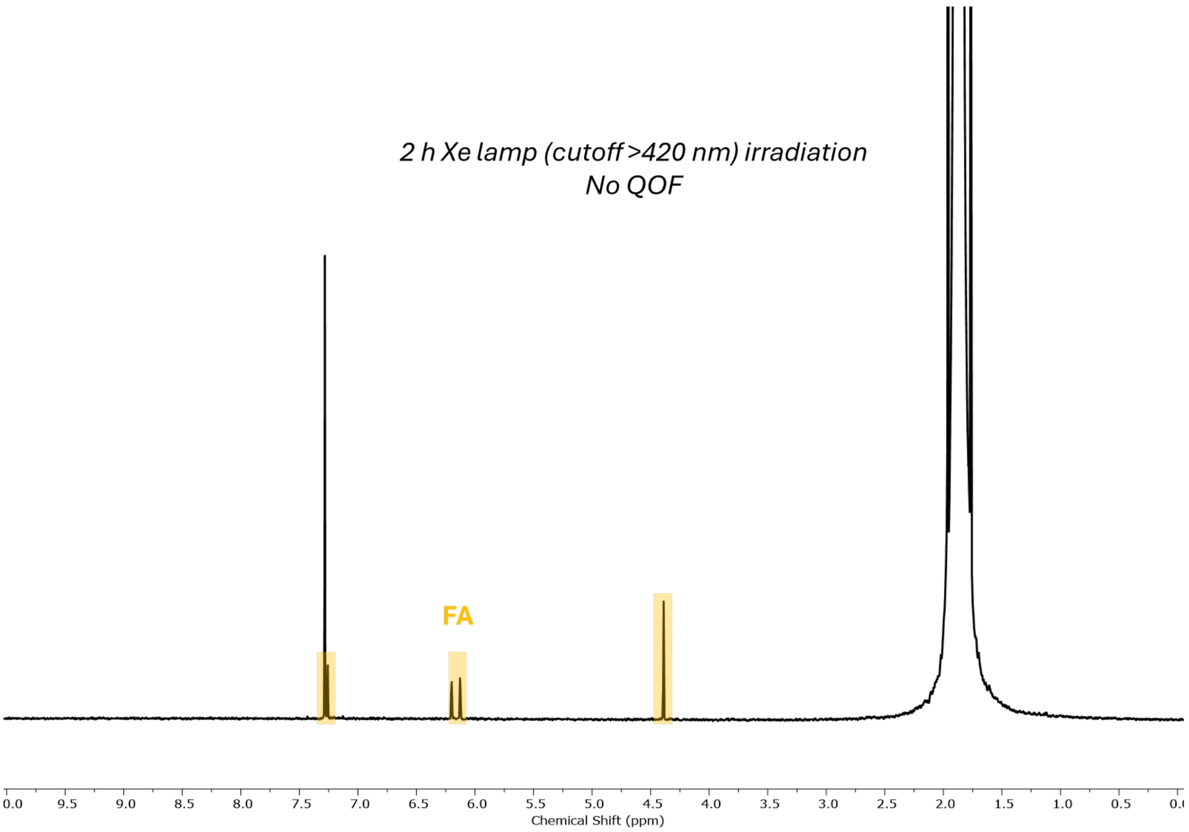

Figure S19: ^{1}H-NMR (300 MHz, $CDCl_3$) spectrum of crude sample from FA photocatalytic oxidation blank experiment in absence of QOF catalyst (30 minutes).

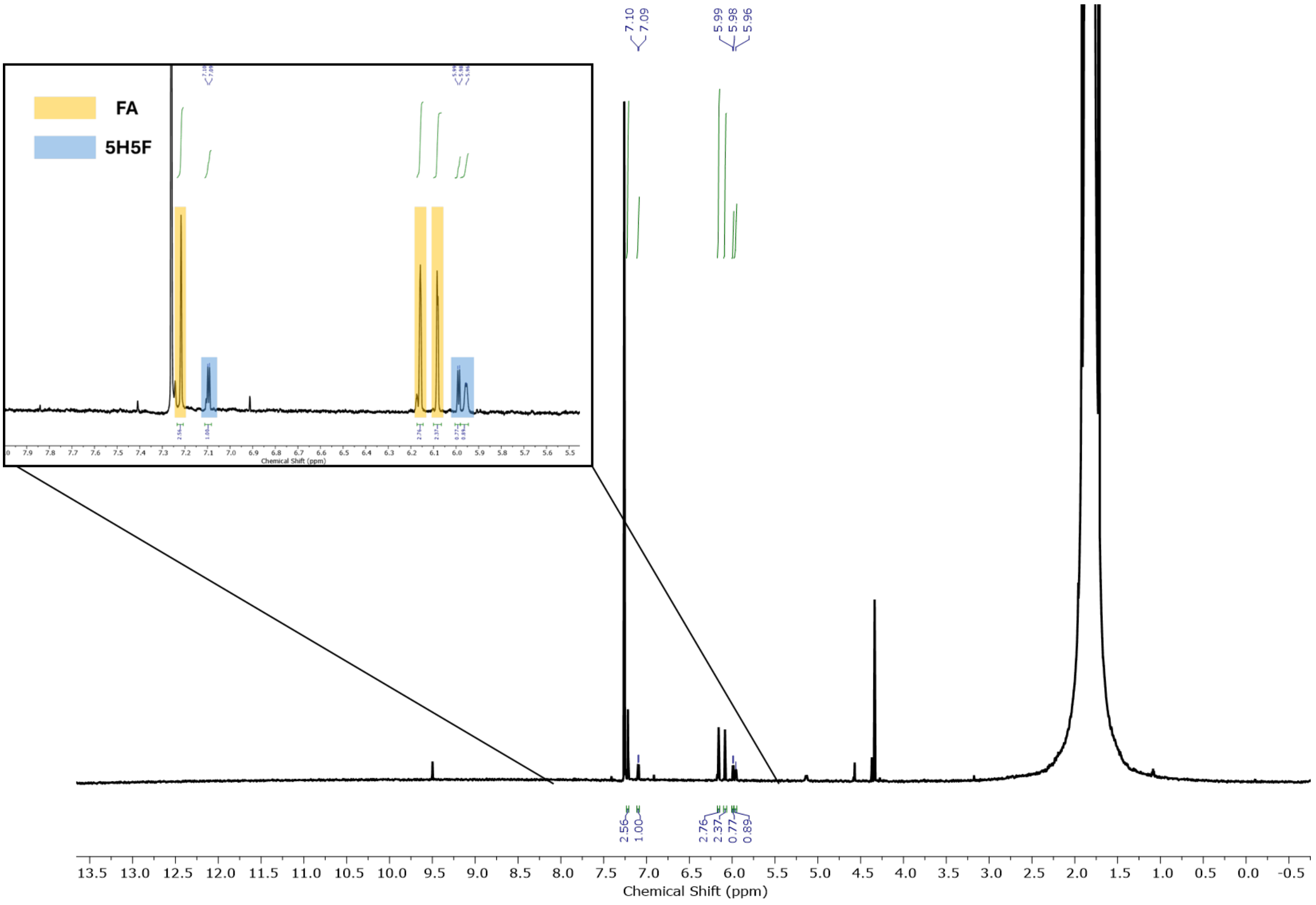

Figure S20: ^{1}H-NMR (300 MHz, $CDCl_3$) spectrum of crude sample from FA photocatalytic oxidation with QOF1 under optimized conditions (2.5 mg of QOF1, 30 minutes).

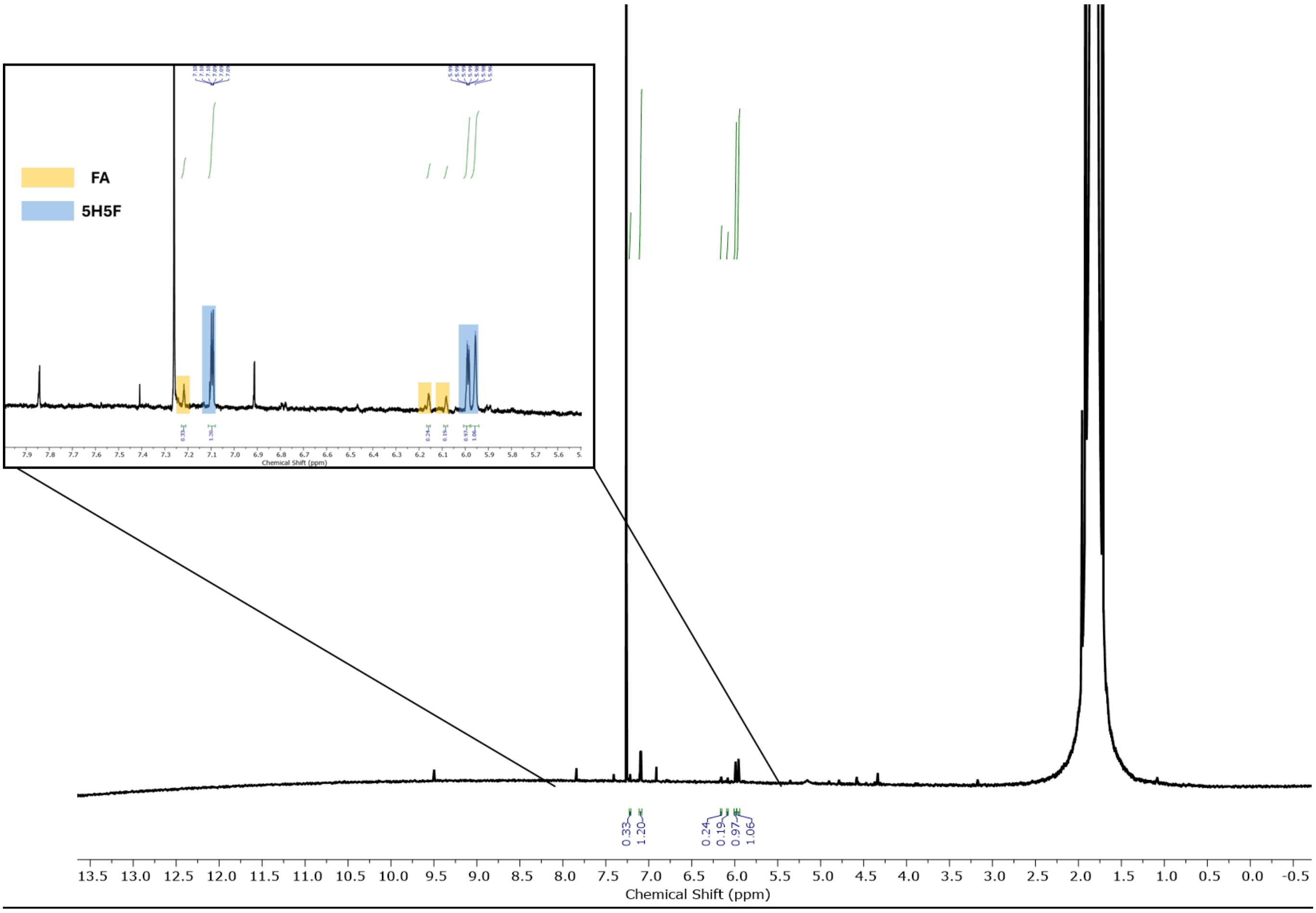

Figure S21: ^{1}H-NMR (300 MHz, $CDCl_3$) spectrum of crude sample from FA photocatalytic oxidation with QOF1-N under optimized conditions (2.5 mg of QOF1-N, 30 minutes).

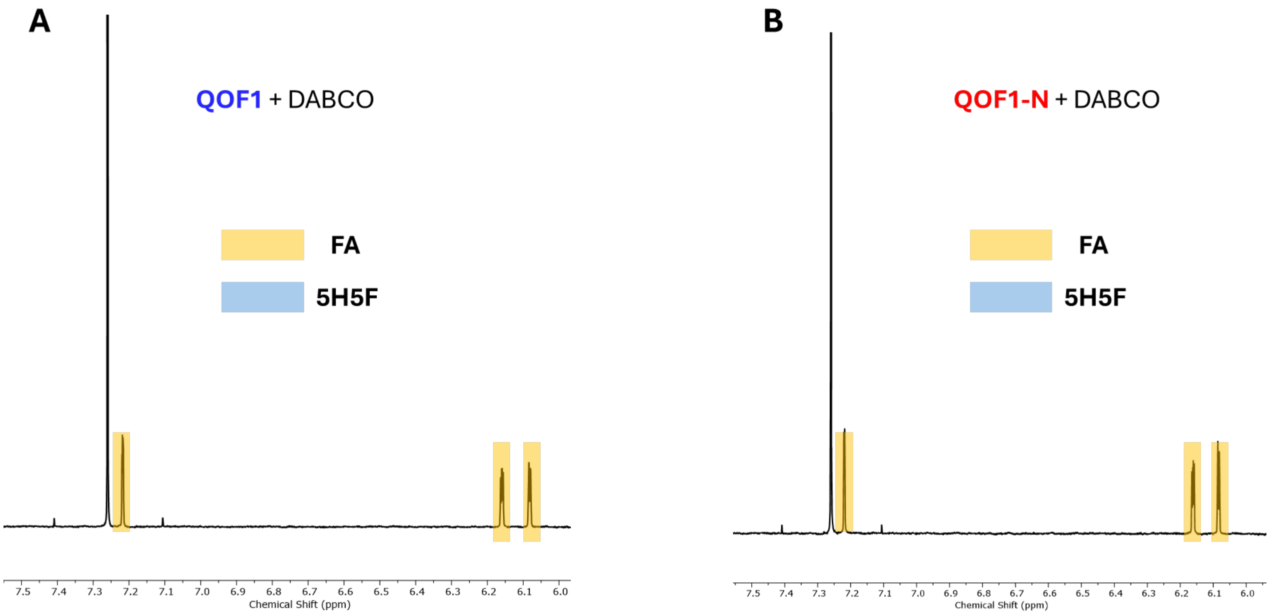

Figure S22: ^{1}H-NMR (300 MHz, $CDCl_3$) spectra of crude sample from FA photocatalytic oxidation in the presence of DABCO under optimized reaction conditions with A) QOF1 and B) QOF1-N.

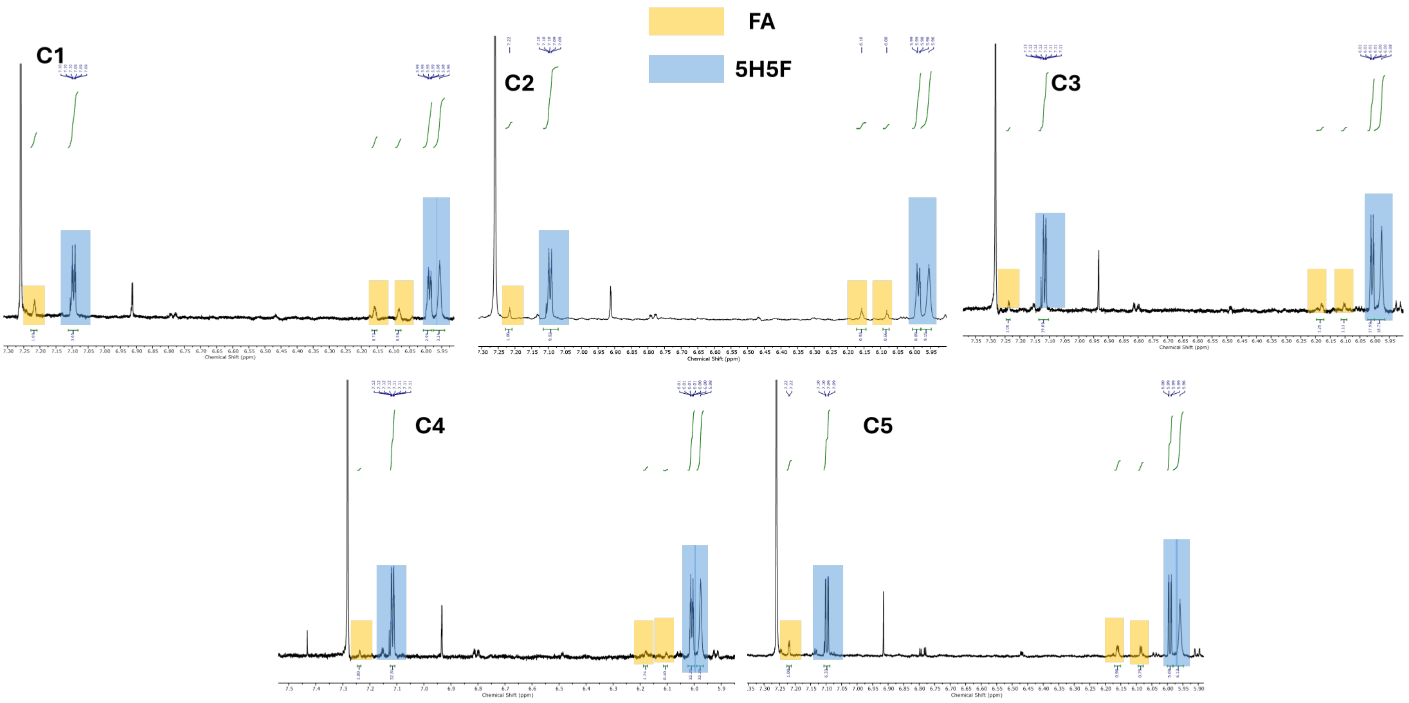

Figure S23: ^{1}H-NMR (300 MHz, $CDCl_3$) spectra of crude sample from each catalytic run (C1-5) of FA oxidation for the recyclability experiments with QOF1-N under optimized conditions (2.5 mg, of QOF1-N, 30min).

19. α-terpinene photocatalytic oxidation experiments

Table S3: Production rates of $^1O_2$ and 5H5F based on α-terpinene oxidation experiments

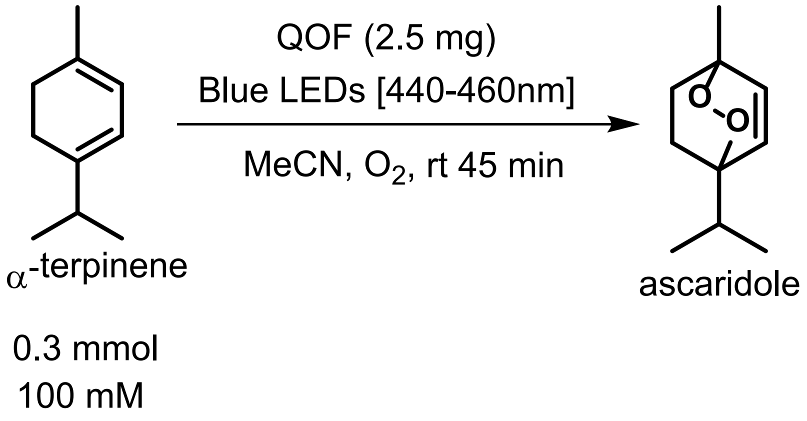

Photocatalyst	Production rate (μmol · mg^{-1}_{QOF}·min^{-1})	
	$^1O_2$ *	5H5F
QOF1	0.44	0.11
QOF1-N	1.04	0.34

* *Estimated from ascaridole formation*

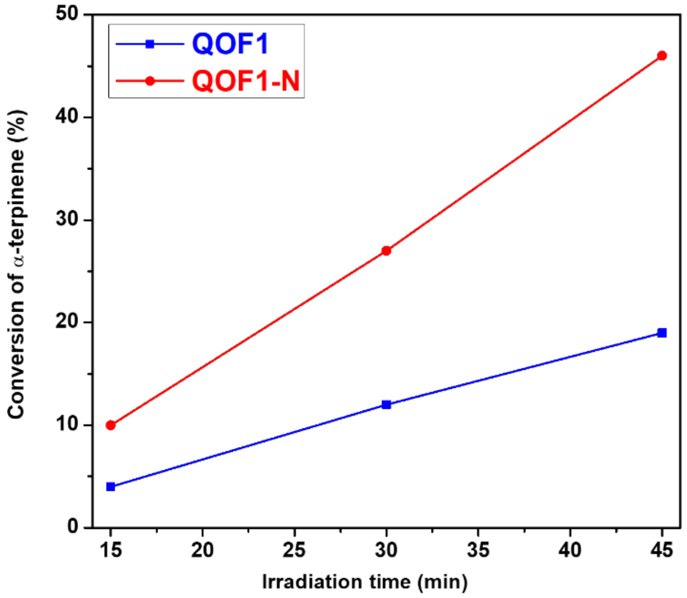

Figure S24: Conversion of α-terpinene during 45 min irradiation with QOF1 (blue) and QOF1-N (red).

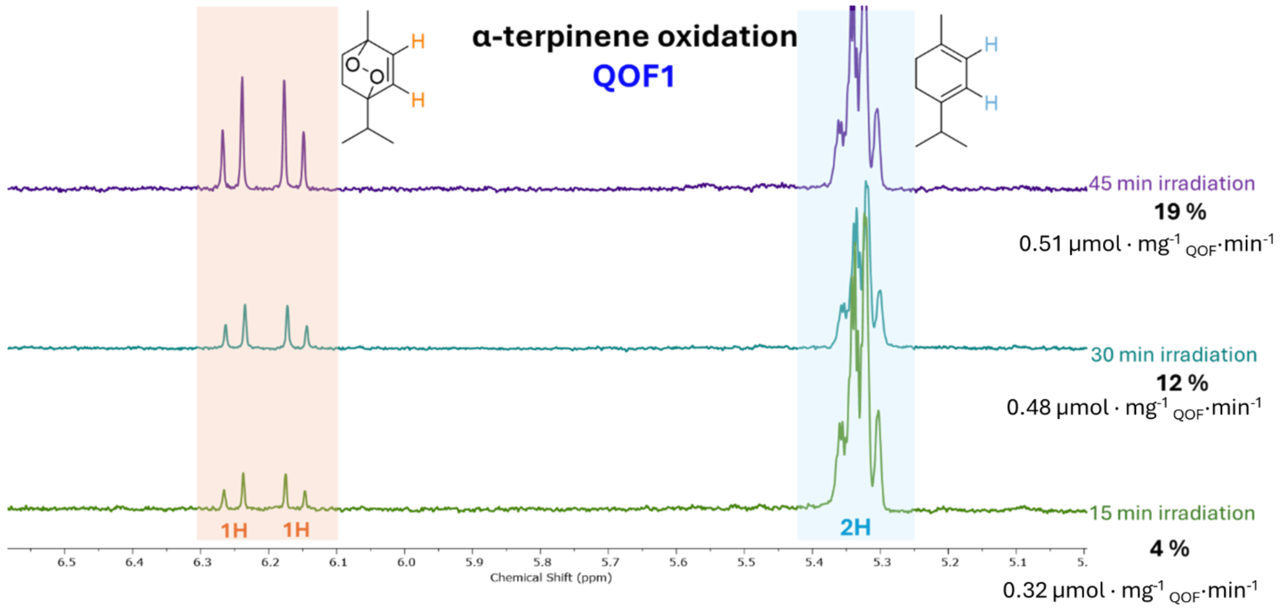

Figure S25: ^{1}H-NMR spectra onset of α-terpinene photocatalytic oxidation by QOF1 at different irradiation times.

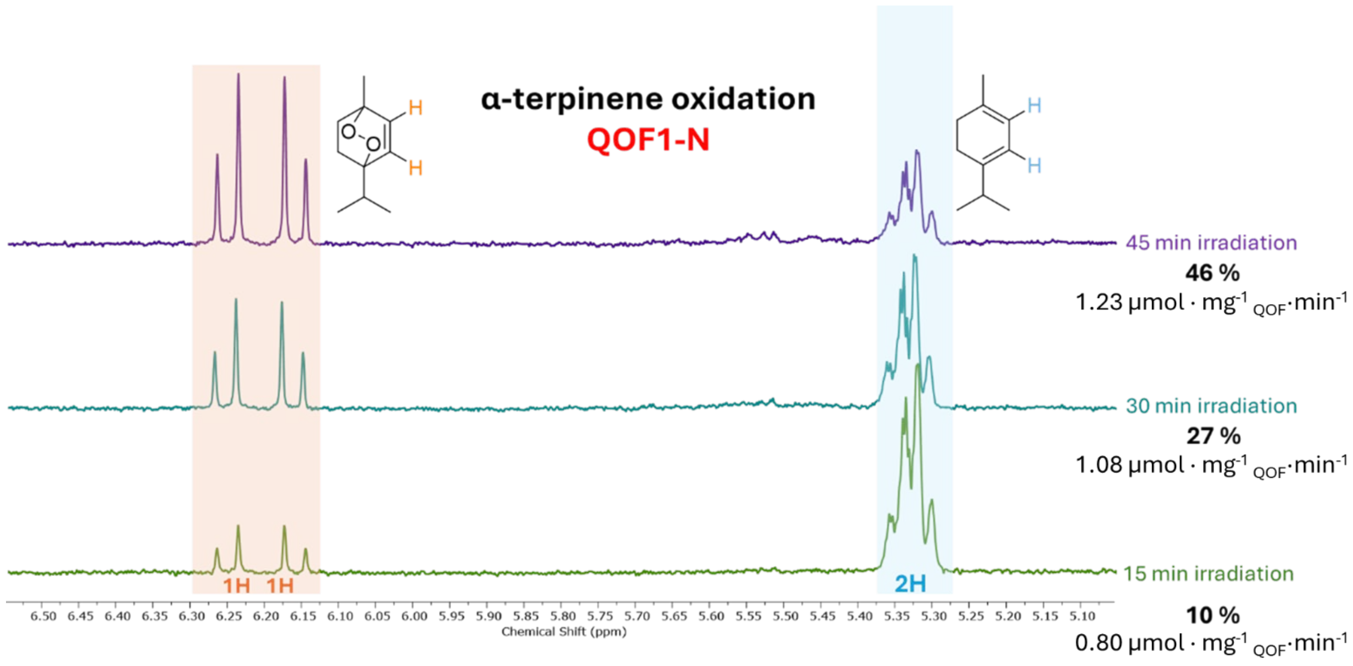

Figure S26: ^{1}H-NMR spectra onset of α-terpinene photocatalytic oxidation by QOF1-N at different irradiation times.

20. Computational models

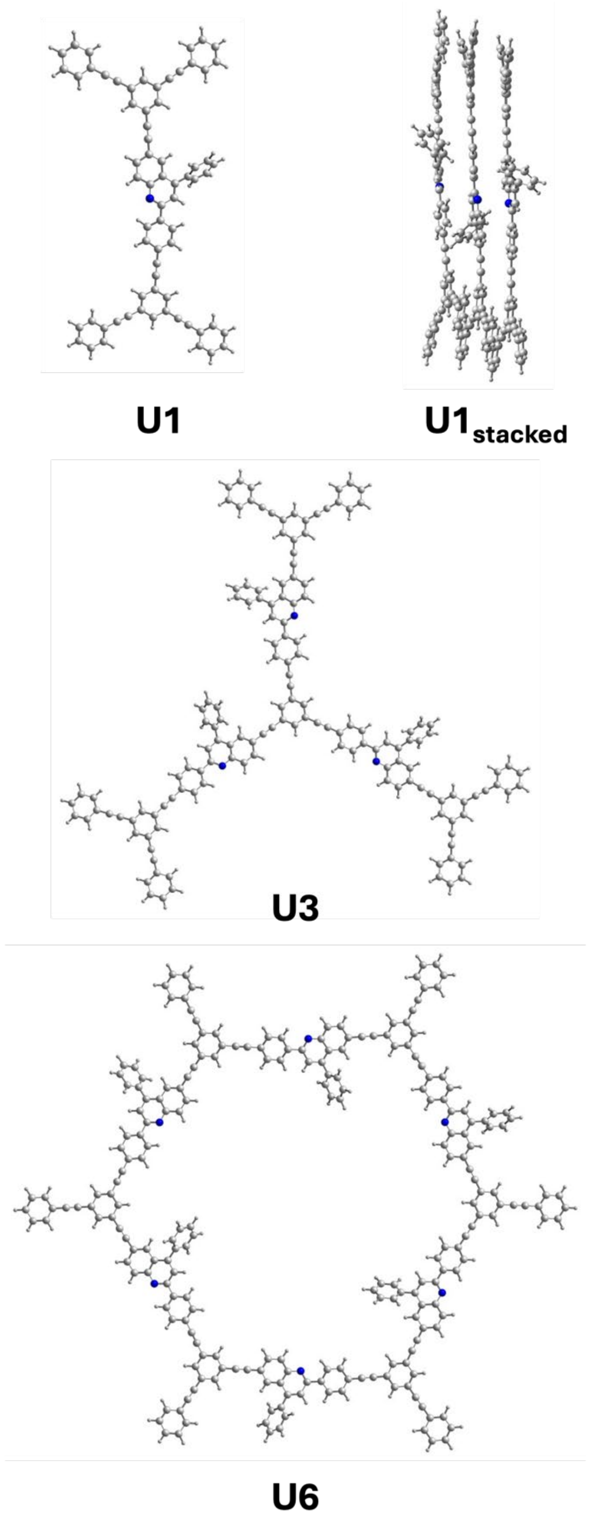

Figure S27: Molecular models used to represent QOF1 and QOF1-N. The labeling indicates the number of quinoline units.

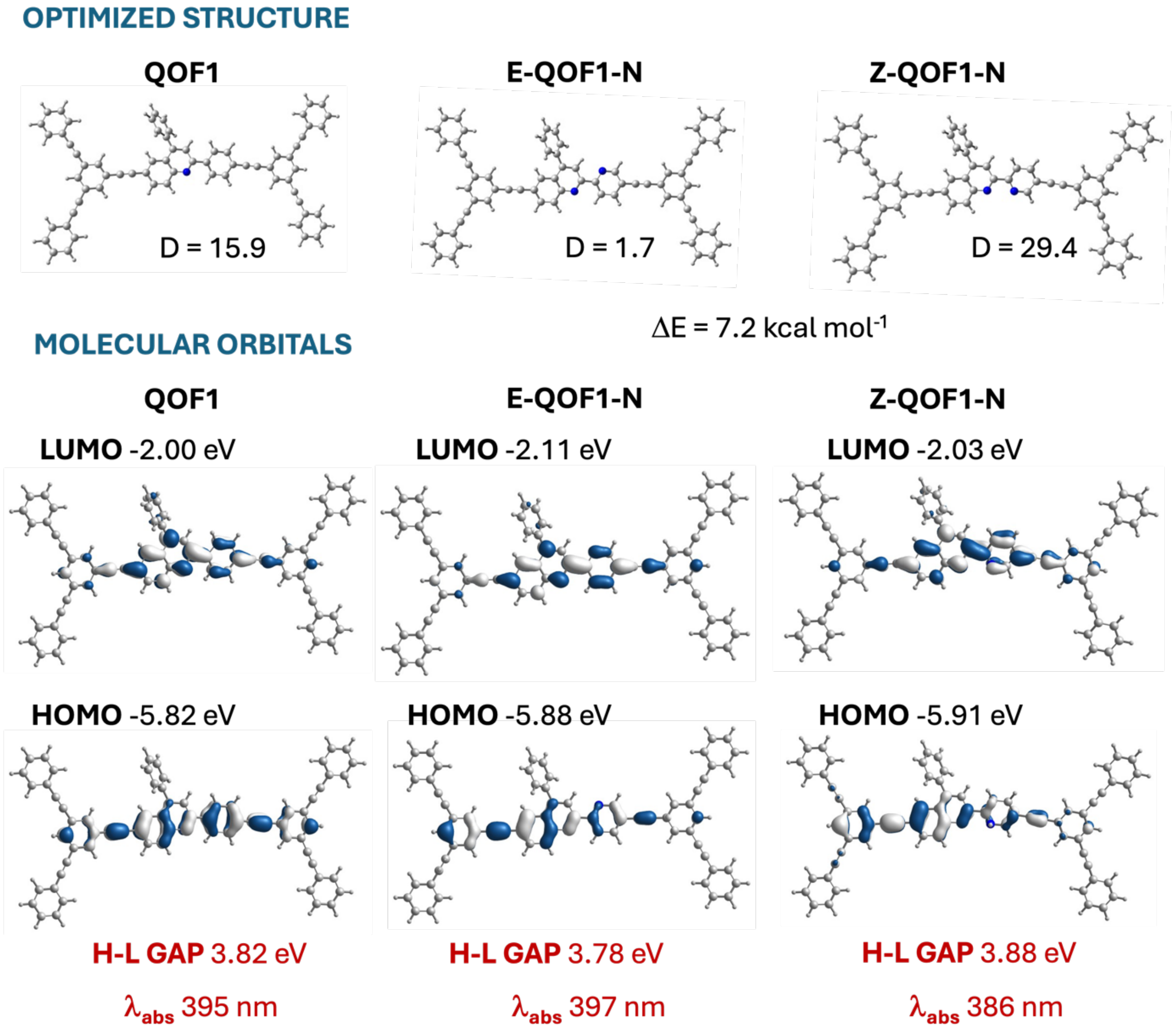

Figure S28: Optimized structures of QOF1 and QOF1-N obtained with the U1 model, and the molecular orbitals involved in the photochemical process.

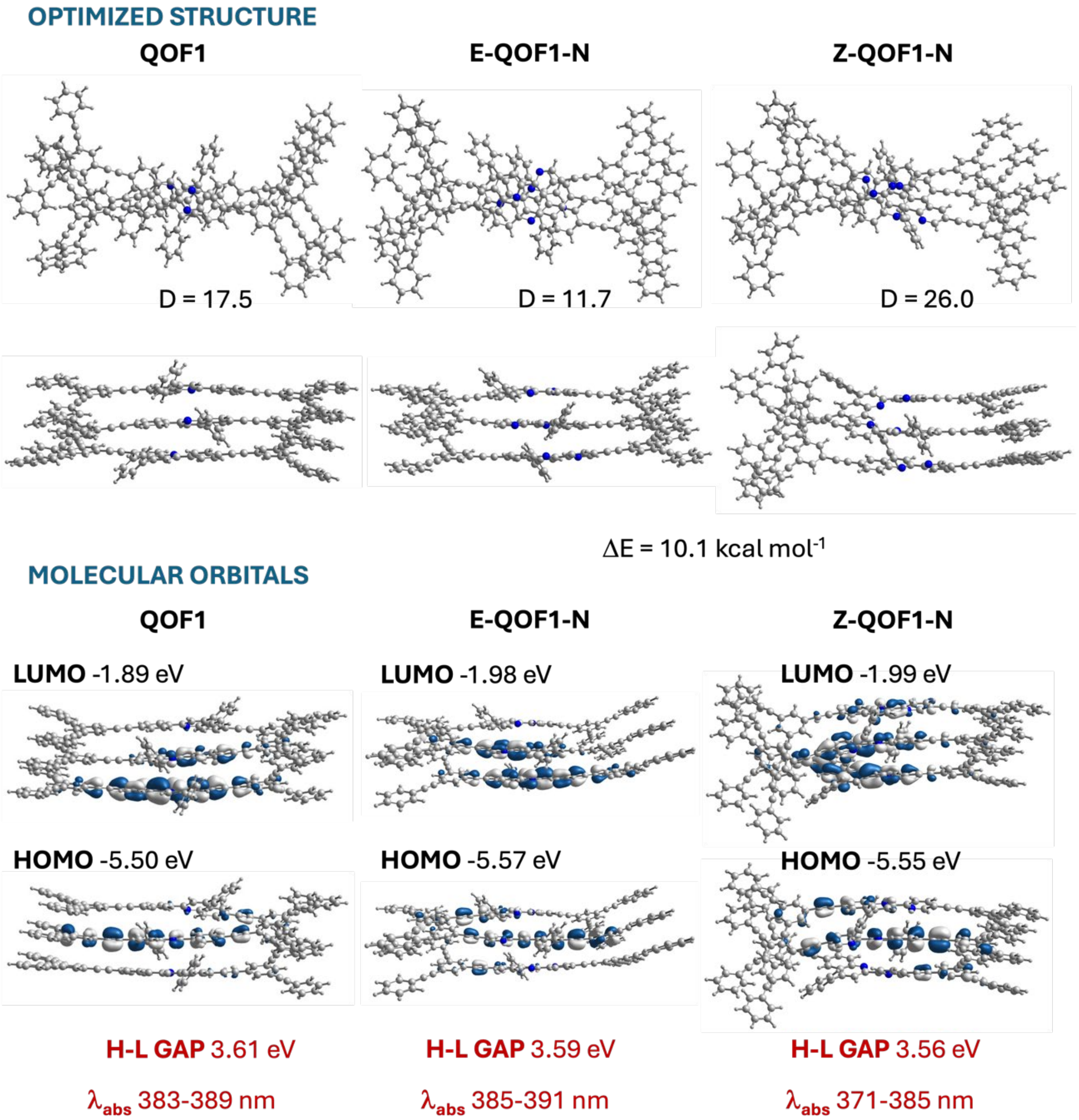

Figure S29: Optimized structures of QOF1 and QOF1-N obtained with the U1 stacked model and the molecular orbitals involved in the photochemical process. The reported dihedral angle corresponds to that of the central unit.

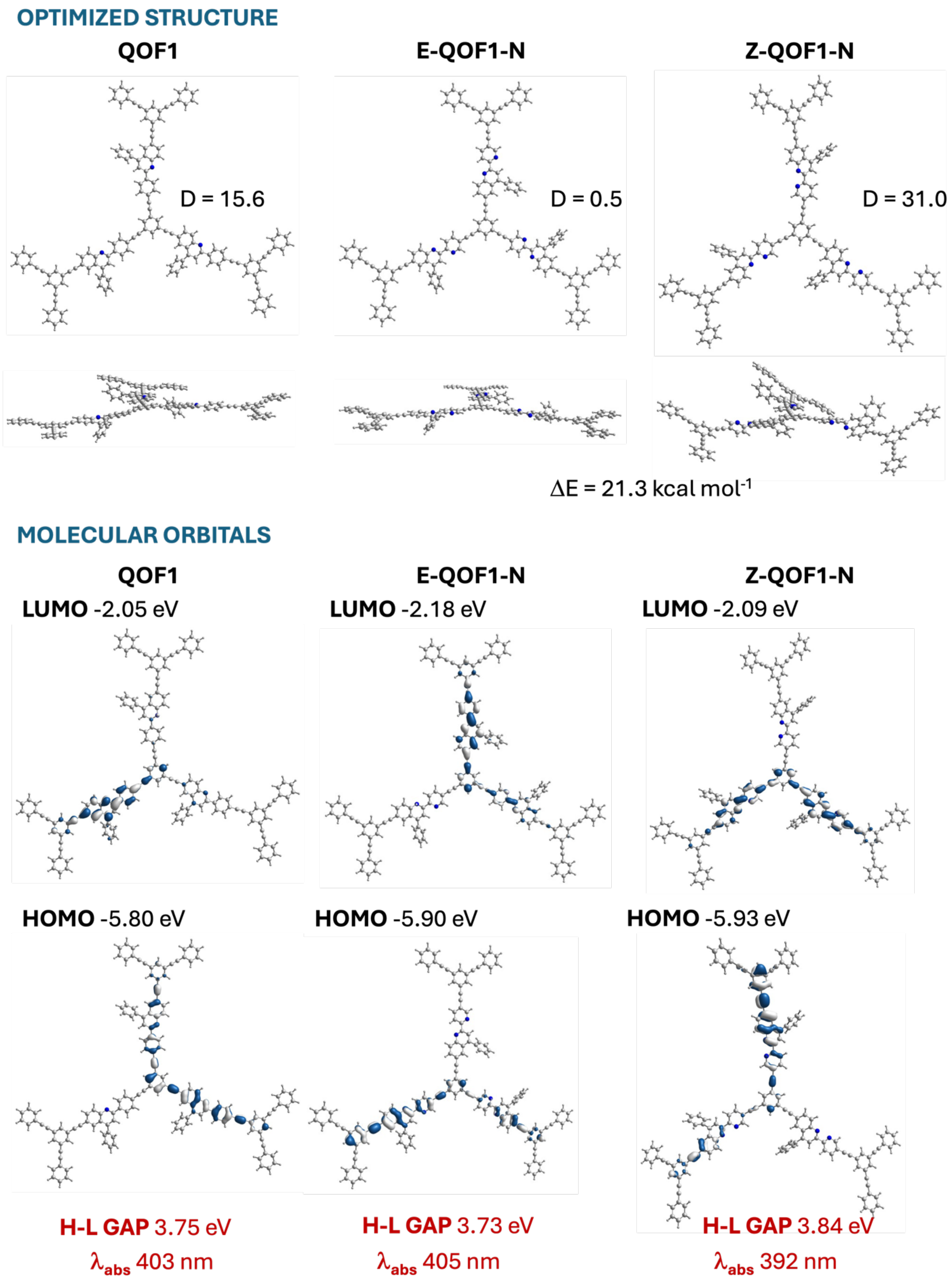

Figure S30: Optimized structures of QOF1 and QOF1-N obtained with the U3 model, and the molecular orbitals involved in the photochemical process.

21. References

1. López-Magano, A.; Solans-Monfort, X.; Salaverri, N.; Marzo, L.; Mas-Ballesté, R.; Alemán, J., Engineering Photocatalytic Porous Organic Materials for Directing Redox versus Energy Transfer Processes. *Solar RRL* **2024,** *8* (2), 2300768.
2. Frisch, M. J.; Trucks, G. W.; Schlegel, H. B.; Scuseria, G. E.; Robb, M. A.; Cheeseman, J. R.; Scalmani, G.; Barone, V.; Petersson, G. A.; Nakatsuji, H.; Li, X.; Caricato, M.; Marenich, A. V.; Bloino, J.; Janesko, B. G.; Gomperts, R.; Mennucci, B.; Hratchian, H. P.; Ortiz, J. V.; Izmaylov, A. F.; Sonnenberg, J. L.; Williams; Ding, F.; Lipparini, F.; Egidi, F.; Goings, J.; Peng, B.; Petrone, A.; Henderson, T.; Ranasinghe, D.; Zakrzewski, V. G.; Gao, J.; Rega, N.; Zheng, G.; Liang, W.; Hada, M.; Ehara, M.; Toyota, K.; Fukuda, R.; Hasegawa, J.; Ishida, M.; Nakajima, T.; Honda, Y.; Kitao, O.; Nakai, H.; Vreven, T.; Throssell, K.; Montgomery Jr., J. A.; Peralta, J. E.; Ogliaro, F.; Bearpark, M. J.; Heyd, J. J.; Brothers, E. N.; Kudin, K. N.; Staroverov, V. N.; Keith, T. A.; Kobayashi, R.; Normand, J.; Raghavachari, K.; Rendell, A. P.; Burant, J. C.; Iyengar, S. S.; Tomasi, J.; Cossi, M.; Millam, J. M.; Klene, M.; Adamo, C.; Cammi, R.; Ochterski, J. W.; Martin, R. L.; Morokuma, K.; Farkas, O.; Foresman, J. B.; Fox, D. J. *Gaussian 16 Rev. C.01*, *Wallingford, CT*, **2016**.
3. Zhao, Y.; Truhlar, D. G., The M06 Suite of Density Functionals for Main Group Thermochemistry, Thermochemical Kinetics, Noncovalent Interactions, Excited States, and Transition Elements: Two new Functionals and Systematic Testing of Four M06-Class Functionals and 12 other Function. *Theoretical Chemistry Accounts* **2008,** *120* (1-3), 215-241.
4. Ditchfield, R.; Hehre, W. J.; Pople, J. A., Self-Consistent Molecular-Orbital Methods. IX. An Extended Gaussian-Type Basis for Molecular-Orbital Studies of Organic Molecules. *The Journal of Chemical Physics* **1971,** *54* (2), 724-728.
5. Hehre, W. J.; Ditchfield, R.; Pople, J. A., Self—Consistent Molecular Orbital Methods. XII. Further Extensions of Gaussian—Type Basis Sets for Use in Molecular Orbital Studies of Organic Molecules. *The Journal of Chemical Physics* **1972,** *56* (5), 2257-2261.
6. Hariharan, P. C.; Pople, J. A., The influence of polarization functions on molecular orbital hydrogenation energies. *Theoretica chimica acta* **1973,** *28*, 213-222.
7. https://www.thermofisher.com/us/en/home/materials-science/learning-center/periodic-table/transition-metal/platinum.html (accessed January, **2025**).
8. Stadnichenko, A.; Svintsitskiy, D.; Kibis, L.; Fedorova, E.; Stonkus, O.; Slavinskaya, E.; Lapin, I.; Fakhrutdinova, E.; Svetlichnyi, V.; Romanenko, A.; Doronkin, D.; Marchuk, V.; Grunwaldt, J.-D.; Boronin, A., Influence of Titania Synthesized by Pulsed Laser Ablation on the State of Platinum during Ammonia Oxidation. *Applied Sciences* **2020,** *10* (14), 4699.
